# Genetic Candidate Variants in Two Multigenerational Families with Childhood Apraxia of Speech

**DOI:** 10.1371/journal.pone.0153864

**Published:** 2016-04-27

**Authors:** Beate Peter, Ellen M. Wijsman, Alejandro Q. Nato, University of Washington Center for Mendelian Genomics, Mark M. Matsushita, Kathy L. Chapman, Ian B. Stanaway, John Wolff, Kaori Oda, Virginia B. Gabo, Wendy H. Raskind

**Affiliations:** 1 Dpt. of Speech and Hearing Sciences, Arizona State University, Tempe, Arizona, United States of America; 2 Dpt. of Communication Sciences and Disorders, Saint Louis University, Saint Louis, Missouri, United States of America; 3 Dpt. of Speech and Hearing Sciences, University of Washington, Seattle, Washington, United States of America; 4 Div. of Medical Genetics, Dpt. of Medicine, University of Washington, Seattle, Washington, United States of America; 5 Dpt. of Biostatistics, University of Washington, Seattle, Washington, United States of America; 6 Dpt. of Communication Sciences and Disorders, University of Utah, Salt Lake City, Utah, United States of America; 7 Dpt. of Environmental and Occupational Health, Environmental Toxicology, University of Washington, Seattle, Washington, United States of America; 8 Dpt. of Psychiatry and Behavioral Sciences, University of Washington, Seattle, Washington, United States of America; NIDCR/NIH, UNITED STATES

## Abstract

Childhood apraxia of speech (CAS) is a severe and socially debilitating form of speech sound disorder with suspected genetic involvement, but the genetic etiology is not yet well understood. Very few known or putative causal genes have been identified to date, e.g., *FOXP2* and *BCL11A*. Building a knowledge base of the genetic etiology of CAS will make it possible to identify infants at genetic risk and motivate the development of effective very early intervention programs. We investigated the genetic etiology of CAS in two large multigenerational families with familial CAS. Complementary genomic methods included Markov chain Monte Carlo linkage analysis, copy-number analysis, identity-by-descent sharing, and exome sequencing with variant filtering. No overlaps in regions with positive evidence of linkage between the two families were found. In one family, linkage analysis detected two chromosomal regions of interest, 5p15.1-p14.1, and 17p13.1-q11.1, inherited separately from the two founders. Single-point linkage analysis of selected variants identified *CDH18* as a primary gene of interest and additionally, *MYO10*, *NIPBL*, *GLP2R*, *NCOR1*, *FLCN*, *SMCR8*, *NEK8*, and *ANKRD12*, possibly with additive effects. Linkage analysis in the second family detected five regions with LOD scores approaching the highest values possible in the family. A gene of interest was *C4orf21* (*ZGRF1*) on 4q25-q28.2. Evidence for previously described causal copy-number variations and validated or suspected genes was not found. Results are consistent with a heterogeneous CAS etiology, as is expected in many neurogenic disorders. Future studies will investigate genome variants in these and other families with CAS.

## Introduction

Children with speech sound disorder (SSD) fall behind their typically developing peers in acquiring speech that is easily understood by others. As extensively reviewed in the literature, signs and symptoms of SSD include distortions, substitutions, omissions, insertions, errors on the syllable or word level, and prosodic errors affecting rhythm and intonation [1–3]. Children with disordered speech have difficulty expressing their thoughts in ways that are easily understood by others [4] and experience negative perceptions on the part of their peers because of their speech differences [5]. Several SSD subtypes have been proposed. One of these is childhood apraxia of speech (CAS), defined as a motor planning or programming disorder affecting the speech production system. The American Speech-Language-Hearing Association (ASHA) issued a position statement regarding CAS (http://www.asha.org/docs/html/PS2007-00277.html) with the following phenotype definition, implicating the central nervous system as the most likely locus of impairment:

Childhood apraxia of speech (CAS) is a neurological childhood (pediatric) speech sound disorder in which the precision and consistency of movements underlying speech are impaired in the absence of neuromuscular deficits (e.g., abnormal reflexes, abnormal tone). CAS may occur as a result of known neurological impairment, in association with complex neurobehavioral disorders of known or unknown origin, or as an idiopathic neurogenic speech sound disorder. The core impairment in planning and/or programming spatiotemporal parameters of movement sequences results in errors in speech sound production and prosody.

The speech of children with CAS may be characterized by some errors commonly seen in children with other forms of SSD but additionally, by unusual errors such as vowel distortions, difficulty initiating or transitioning between articulatory gestures, lack of differentiation between stressed and unstressed syllables or mis-stressing syllables, distorted substitutions, syllable segregation (resulting in a staccato-like rhythm), schwa insertions, voicing errors, slow rate, slow diadochokinetic rates, and/or increased difficulty with multisyllabic words [6]. Compared to other subtypes of SSD, CAS is considered to be more severe, requiring intense and specialized treatment [7, 8]. Children with CAS are at increased risk for reading/spelling disorders [9–11]. According to one estimate, CAS is diagnosed in .01% to .02% of children in the United States [12].

Disordered speech consistent with CAS can be part of syndromes of genetic etiology. In one large multigenerational family referred to as the KE family, disruptions in the *FOXP2* gene (OMIM #605317) on chromosome (chr) 7 caused a severe speech disorder in the presence of nonverbal oral dyspraxia and disordered language [13–15]. Structural and functional brain changes were observed as well, characterized by reduced grey matter density in the caudate nucleus, cerebellum, and inferior frontal gyrus [16] and reduced activation during a nonword repetition task in the premotor, supplementary, and primary motor cortices and in the cerebellum and basal ganglia [17]. A functionally related gene, *CNTNAP2*, plays a role in language [18] and reading [19] ability. Approximately 18% of children with galactosemia (OMIM #230400, OMIM #606999), a metabolic disease caused by mutations in the *GALT* gene (OMIM #606999) on 9p13.3 [20], exhibit signs of CAS [21]. Variants in the *ELP4* (OMIM #606985) and *PAX6* (OMIM #607108) genes on 11p13 have been associated with Rolandic epilepsy, which is frequently accompanied by disordered speech consistent with CAS [22]. Duplications of a region on 7q11.23 are associated with developmental delays, characteristic facial anomalies, social anxieties, and severe delays in language and speech abilities, the latter consistent with CAS [23, 24]. In five of nine individuals with subtelomeric or interstitial 12p13.33 deletions and speech delays, the speech phenotype was consistent with CAS [25]. In a child with a severe speech disorder characterized by apraxic traits as well as muscle weakness, we found a *de novo* heterozygous deletion of the *BCL11A* gene (B-cell CLL/lymphoma 11A, OMIM #606557) on chr 2 [26], located within a larger microdeletion region associated with global deficits in motor development and muscle tone as well as growth retardation, intellectual disability, absence of verbal communication, and/or craniofacial and skeletal dysmorphic features [27–34]. Our case study suggests that *BCL11A* plays a role in aspects of motor planning/programming and muscle tone required for speech.

There is evidence that in some cases, idiopathic CAS has a genetic etiology, but causal genes have not yet been validated. In studies of three individuals with CAS, evidence from duplicated or deleted DNA regions pointed to 16p11.2 as a candidate region [35, 36]. In a study of 24 unrelated children with CAS, 12 had copy-number variations (CNVs) on ten different chromosomes; findings included one 16p11.2 deletion [37]. One of the children had a *FOXP2* mutation, and three of the CNV regions contained other candidate genes. In an exome variant study in 10 unrelated individuals with CAS, variants of interest were found on chrs 3, 6, 7, 9, and 17, where some participants had more than one of the variants [38]. Potentially deleterious variants were reported in genes suspected to cause CAS (*FOXP1*, OMIM #605515, *CNTNAP2*, OMIM #604569) and genes associated with phenotypes frequently co-occurring with CAS (*ATP13A4*, OMIM #606693, *CNTNAP1*, OMIM #602346, *KIAA0319*, OMIM #609269, and *SETX*, OMIM #608465).

In two multigenerational families with familial nonsyndromic CAS, we showed that the speech phenotype was associated with oral and hand motor deficits, especially when the tasks required temporal integration of alternating-sequential movements. In the oral motor domain, diadochokinetic (DDK) rapid repetition of multisyllabic tokens (/pata/, /taka/, and /pataka/) was used to assess alternating-sequential functioning and the analogous hand task was rapidly tapping two computer keys using two fingers in an alternating fashion. Less impaired was performance on tasks requiring repetitive movement sequences in the oral domain (/pa/, /ta/, and /ka/) and single key tapping in the hand domain [39]. For a parametric genome-wide linkage analysis in one of these participating families, we used a measure of alternating-sequential DDK ability obtained by subtracting standard scores of performance on monosyllabic syllable repetition from standard scores of performance on multisyllabic syllable repetition as the input variable, with a maximum possible LOD score of 1.78. Two new regions of interest were found, one on 7q36.1-q36.3 (bp 143,723,666–159,138,663; LOD = 1.35) and one on 6p21.2-p12.3 (bp 36,632,927–64,590,642; LOD = 1.10) [40]. The 6p region overlapped with a recently identified region of interest for dyslexia [41].

Together, these findings are consistent with a heterogeneous CAS etiology. Here, we posit that speech development is complex and can be influenced by several genetic and environmental factors with varying levels of impact in the same individuals. Discovery of genetic risk factors of high impact may be more successful in families than in unrelated individuals because these factors are likely to be shared by affected members of the same family.

We recently described phenotypic aspects in a multigenerational family, here referred to as “Family A”, with familial CAS [42]. Most of the individuals with current or past CAS did not produce their first word until age 3 years whereas first words typically emerge around the first birthday. Their speech was difficult to understand by others until they reached age 5 to 7 years, a milestone typically reached by age 4 years. Most of the affected family members required a minimum of three years of speech therapy to acquire intelligible speech. Performance on tasks with high sequential processing loads including multisyllabic DDK testing [43, 44], nonword imitation [45–47], rapid automatic naming [48], nonword decoding [49, 50], and spelling [51] differentiated between family members with and without a history of CAS, whereas there were no group differences in tasks with low sequential processing loads. A qualitative analysis of errors during real word and nonword imitations showed that the adults with a history of disordered speech produced more phoneme sequencing errors, compared to those without such a history. These findings were interpreted as consistent with a deficit in sequential processing that was not limited to motor programming but also manifested in linguistic and cognitive tasks. Results were replicated in adults from five other families with familial SSD including CAS [52].

The affectation pattern in Family A is consistent with a genetic etiology of CAS. The purpose of the present study, hence, was to investigate this hypothesis using a set of complementary methods. Similar methods were used to investigate the same hypothesis in a second multigenerational family with familial CAS, here referred to as Family B, and results were compared.

## Materials and Methods

### Participants and Behavioral Measures

This study was conducted with the approval of the University of Washington’s institutional review board. Adults gave written consent, parents gave written permission for their minor children to participate in the study, and additionally, school-age children gave written assent and preschool-age children gave oral assent. Extensive family history interviews were conducted with the participating adults in each family to obtain background information regarding presence of an SSD diagnosis and history of speech therapy services for the interviewed persons themselves as well as other family members. In addition, each adult filled out a questionnaire regarding her/his educational, developmental, and health history. Parents provided details regarding the developmental history of each of their children. Copies of any available written assessment reports were obtained. Affectation status was assigned based on this information and, for young children who had not yet been professionally assessed for the presence of SSD, additionally on performance on standardized and nonstandardized speech measures. In a few cases where sufficient evidence was not available, unknown affectation status was assigned.

**Fig 1:**
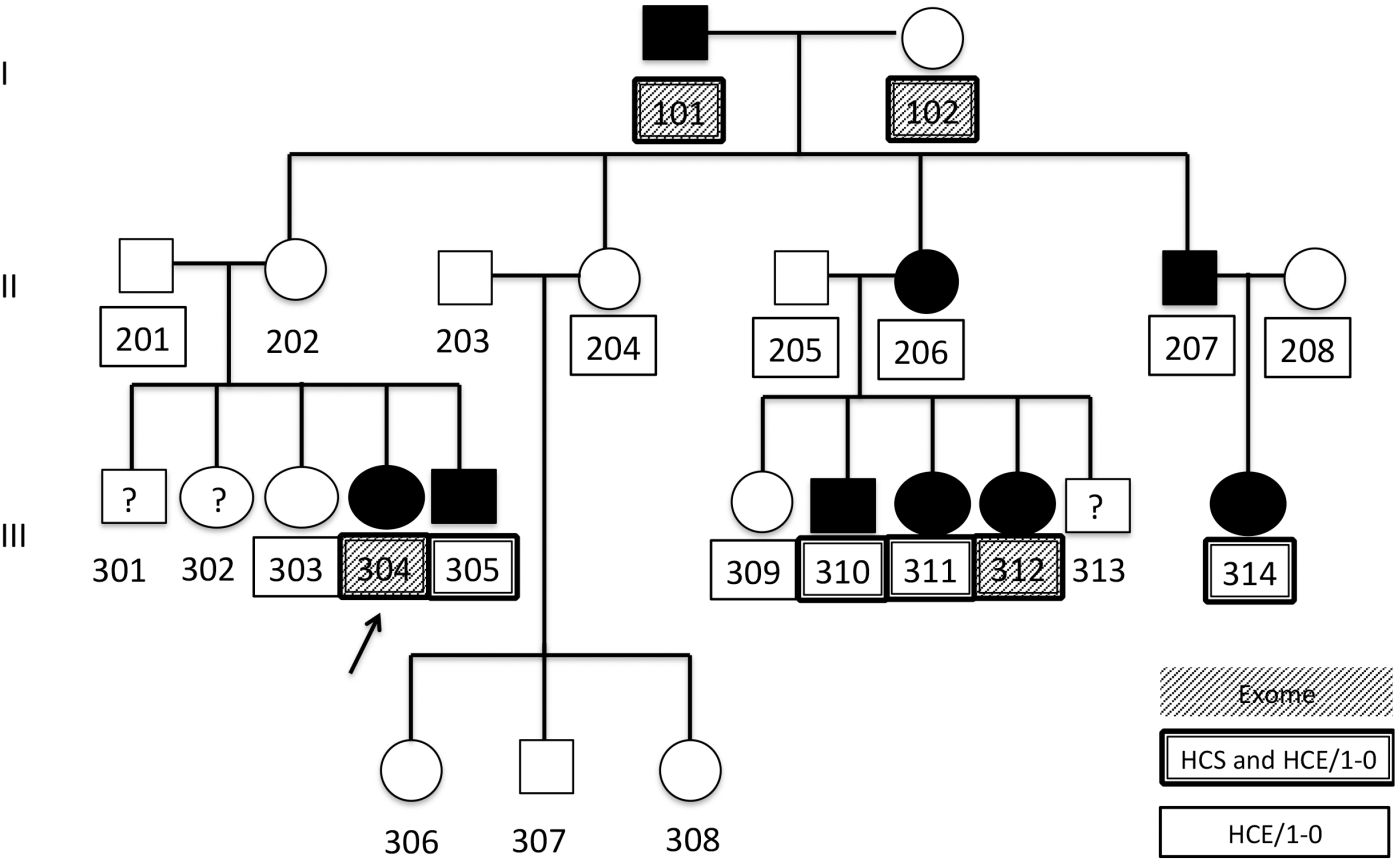
Family diagram for Family A. Square shape = male, circle shape = female, black fill = affected, white fill = unaffected,? = affectation status unknown, arrow = proband, HCS = Illumina HumanCytoSNP-12v2, HCE/1-0 = Illumina HumanCoreExome-12v1-0_B. Numbers underneath each symbol are individual IDs. Boxes around an ID identify individuals with SNP array data. Filled boxes indicate IDs that also have whole exome sequence data.

Family A consists of 24 members in three generations with a familial SSD consistent with CAS (Fig 1; note that the text refers to individual ID numbers with the family identifier as a prefix for clarity). All participants are of European descent, with a small admixture of Japanese descent in six of the participants. Phenotypes and DNA were available for two founders, four adult offspring and their spouses, and 13 grandchildren, 11 of whom could be classified with respect to CAS affectation. The oldest grandchild, A-301, was unable to contribute DNA or participate in the testing; only his developmental history was available. The proband, ID A-304, age 10 years at the time of testing, had a history of severe CAS requiring intense and prolonged speech therapy. The grandfather, ID A-101, reported receiving speech services as an elementary school student whereas the grandmother, A-102, did not report receiving such services. Both grandparents reported individuals biologically related to them with difficulties in the area of speech and language acquisition. No written records were available regarding the grandparents’ speech development. Two of their four participating adult offspring (A-206, A207) had received speech services for five or more years during their early elementary and middle school years. Of the 14 grandchildren, four (A-304, A-305, A-310, A-311) had previously been given a diagnosis of CAS and were currently receiving speech therapy or had completed their course of speech therapy, two (A-312, A-314) were diagnosed based on the speech testing conducted as part of this study, two (A-301, A302) had been diagnosed with a mild speech delay not consistent with CAS as preschoolers, one was too young (15 months) to be diagnosed unambiguously, and five had never received an SSD diagnosis of any type. Details regarding the behavioral findings have been reported previously [42].

**Fig 2:**
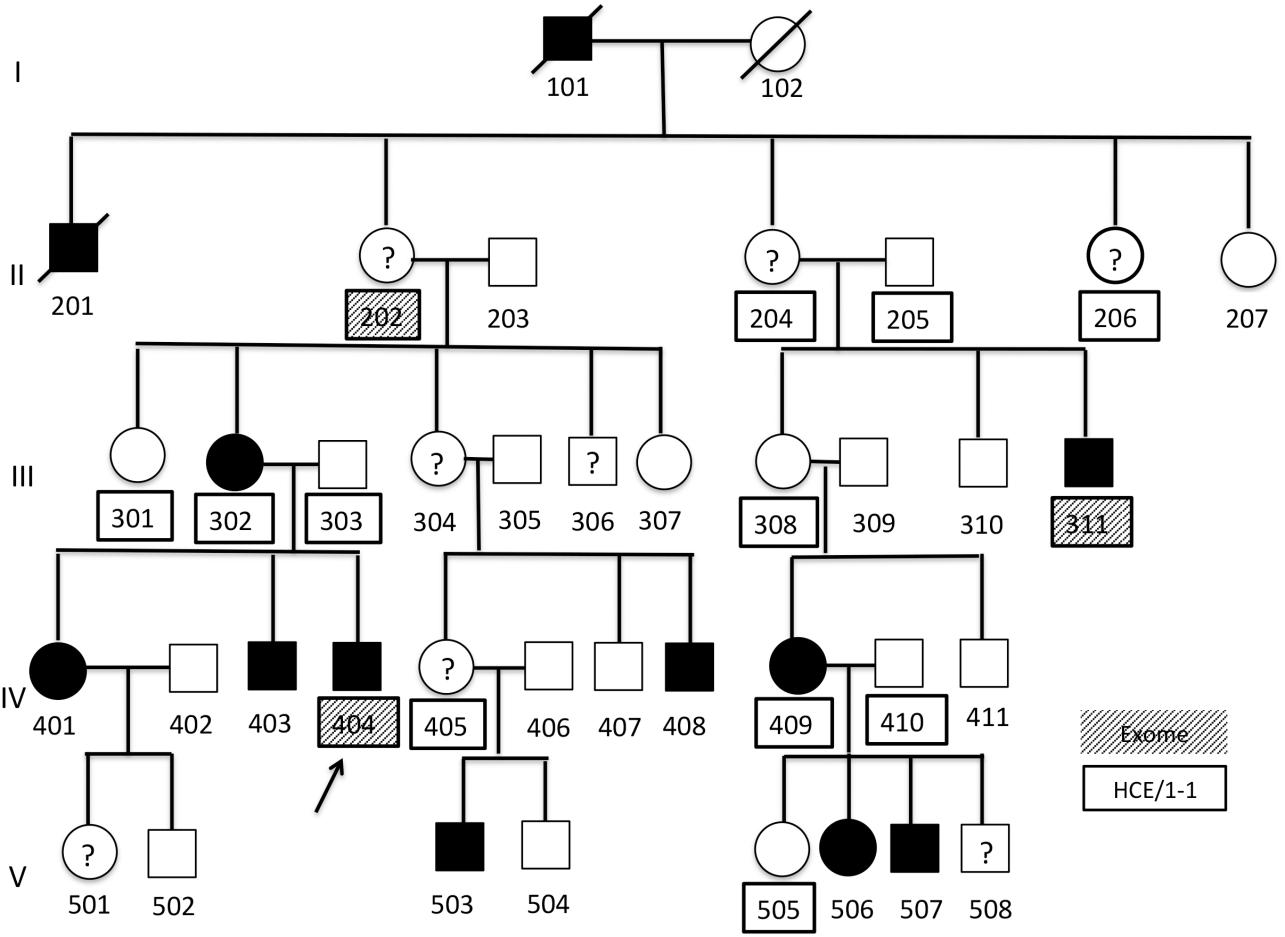
Family diagram for Family B. Square shape = male, circle shape = female, black fill = affected, white fill = unaffected,? = affectation status unknown, arrow = proband, HCE/1-1 = Illumina HumanCoreExome-12v1-1_B. Numbers underneath each symbol are individual IDs. Boxes around an ID identify individuals with SNP array data. Filled boxes indicate IDs that also have whole exome sequence data.

Family B also has a history of familial CAS. The family consists of 39 members in five generations, all of European descent except for six individuals with an admixture of African American descent (Fig 2). DNA was available for 14 participants (B-202, B-204, B-205, B-206, B-301, B-302, B-303, B-308, B-311, B-404, B-405, B409, B-410, B-505). Questionnaire and interview information was available for these participants and also for B-506 and B- 507. All of these participants except B-206, B-405, and B-410 participated in behavioral testing.

The proband, B-403, was 14 years old at the time of testing. He was born at term after an uncomplicated pregnancy and delivery and passed regularly scheduled health, vision, and hearing checks throughout the preschool years. He began receiving speech and expressive language services at age 2;5 due to severe delays in these areas. His diagnosis of CAS at this time was based on severely impaired articulation skills in the presence of severe oral apraxia, not further described in the assessment report. At age 3;8, speech testing with the Structured Photographic Articulation Test II (SPAT-II) [53] resulted in a standard score of 66 (population mean = 100, SD = 30; 1^st^ percentile, far below normal limits), consistent with a severe SSD. His consonant inventory was extremely restricted, consisting of only /d, b, m, n/. Oral motor testing showed deficits in imitating tongue movements. Language testing using Clinical Evaluation of Language Fundamentals-Preschool (CELF-P) [54] showed an Auditory Comprehension standard score of 95 (37^th^ percentile, within normal limits) and an Expressive Comprehension standard score of 50 (1^st^ percentile, far below normal limits). At age 5;8, the proband underwent an occupational therapy evaluation that revealed severe fine motor deficits, especially in grasping and eye-hand coordination skills, qualifying him for services in this area, whereas his gross motor development was found to be within normal limits. Upon entering school, the proband showed difficulty with reading and spelling. For instance, at age 8;10, when tested with the Woodcok-Johnson Tests of Achievement III [55], he obtained a standard score of 63 in Broad Written Language and 64 in Broad Reading (both 1^st^ percentile). The ASHA technical report on CAS lists a small consonant inventory, poorer expressive than receptive language skills, oral apraxia, fine motor deficits, and difficulty with written language as frequently co-occurring conditions (http://www.asha.org/docs/html/PS2007-00277.html). Testing at age 14 showed severe difficulty with nonword imitation [45–47], especially in the form of rearranged phoneme sequences. During diadochokinetic testing, his syllable durations for the monosyllables /pa/, /ta/, and /ka/ were longer than expected for his age, indicative of slow syllable production speed (z = -1.11, -1.84, and -1.13, respectively), but excessively long for the trisyllable /pataka/ (z = -4.32), indicative of severe difficulties with motor planning of complex sequences. Increased difficulty with multisyllabic diadochokinetic tasks, compared to monosyllabic tasks, was reported in our previous studies of children and adults with CAS histories [39, 42, 52, 56].

A similar history of SSD and delays in written language was also reported by his mother, B-302, and another relative, B-311, whereas B-409 reported a history of SSD only in the absence of difficulties with written language, and B-405, a history of difficulties with written language in the absence of SSD. Two family members, B-101 and B-201, both deceased, were reported to have had severely disordered speech during childhood but written records were not available for them or any other members in generations I and II.

Because of her concerns that the severe speech disorder could be of genetic origin, the proband’s mother had sought genetic testing for the proband and herself five years prior to participating in this study. According to the clinical report, a microarray analysis of 622 loci using 1,887 BAC clones was performed on DNA derived from peripheral blood. Two interstitial duplications, separated by a normal intervening sequence, were detected on 15q26.3 ([CTD-3210F22, RP11-947PI-631H11]x3, [RP11-262p8, RP11-654A16]x2, [RP11-20G13, CTD-3221M10]x3), summing to 2 Mb in size. The centromeric duplication contains the entire *FAM169B* gene and the telomeric one, part of the *MEF2A* gene. Fluorescence in situ hybridization (FISH) analysis using two BAC clones from the two regions (CTD-3210F22, RP11-20G13) showed a pattern consistent with duplication. The same duplication was also found in the mother’s DNA using microarray analysis. The clinical significance of this abnormality could not be determined at the time of the clinical report.

Family A provided more direct phenotypic observations and fewer missing samples, compared to Family B. Therefore, the main focus of this study was placed on Family A and data from Family B were used for purposes of comparison.

### Genetic and Statistical Methods

#### Overview

Complementary genomic approaches were selected because the genetic etiologies of CAS cases in the literature to date include not only a point mutation [13, 14, 37] but also deletions and duplications [26, 35–37]. To investigate the presence of single, relatively rare alleles in the families, we conducted linkage analysis. To detect duplications and/or deletions, we performed copy-number variation (CNV) analysis. Identity-by-descent (IBD) analysis was used to investigate more common segregating variants in Family A, where the grandfather had received speech therapy during childhood but the possibility of childhood speech difficulties in the grandmother could not be ruled out completely. Whole exome sequencing (WES) followed by variant filtering was performed in both families to identify candidate variants. Because of greater statistical power to detect linkage in Family A, compared to Family B, selected candidate variants were genotyped and checked for segregation in Family A only.

#### Genotyping and Sequencing

DNA was extracted from peripheral blood using standard laboratory procedures. The samples passed quality control checks for sample swaps and incorrectly specified parentage. Because of the phenotypic overlaps with the previously described KE family where a point mutation in the *FOXP2* gene caused a severe speech and language disorder [13, 14], this gene was ruled out by exclusion mapping [57] prior to genome-wide analysis procedures.

The University of Washington (UW) Center for Mendelian Genomics (CMG) provided single nucleotide polymorphism (SNP) genotypes based on three arrays, as well as WES. In Family A, genotypes for eight participants (Fig 1) were obtained using the Illumina HumanCytoSNP-12v2 array (henceforth HCS) with 298,563 markers. Genotypes for these and eight additional participants (Fig 1) were obtained using the Illumina HumanCoreExome-12v1-0_B array (henceforth HCE/1-0) with 538,448 markers. In Family B, all 14 available DNA samples (Fig 2) were genotyped using the Illumina HumanCoreExome-12v1-1_B (henceforth HCE/1-1) with 542,585 markers.

In Family A, DNA samples from two cousins, ID A-304 and A-312, both with a diagnosis of CAS and highly informative based on position in the family pedigree, and samples from the two grandparents were selected for WES (Fig 1). Similarly, B-202, B-311, and B-404 were selected for WES in Family B (Fig 2). Following methods previously described in detail [58], the NimbleGen in-solution SeqCap EZ Exome Library v2.0 (Roche, Basel, Switzerland) was used to capture the exome and adjoining regions, following the manufacturer’s instructions. Short-read sequencing was done on an Illumina HiSeq 2000 platform.

For Family B, to evaluate whether the previously reported duplications on 15q26.3 segregated with the disorder, one probe within each of the duplicated regions (Hs02820990_cn, located within *FAM169B* at bp 98,981,473, and Hs01667266_cn, located within *MEF2A* at bp 100,250,891), and two control probes (Hs03312008_cn at bp 97,806,447 and Hs05387770_cn at bp 101,256,220) were typed in seven strategically selected samples.

#### Statistical Analyses

Prior to the SNP-based linkage analyses in the two families, power analysis with 1,000 simulations was conducted using the SLINK package [59, 60]. Under the assumed model of autosomal dominant inheritance, there was one case of nonpenetrance in Family A (A-202) and one in Family B (B-308). As in other genome-wide family-based studies with similar mode of inheritance and evidence for reduced penetrance [61], we assumed parameters of penetrance = 0.50 in the two high-risk genotypes and 0.01 in the low-risk genotype. A simple reduced penetrance model similar to this that allows for sporadic cases works well in situations where the penetrance is unknown but incomplete, outside information to inform the parameters further is not available, and the genotype-phenotype relationship is likely to have at least some complexity [62]. In Family A, the resulting maximum log odds (LOD) score in the power analysis at theta = 0 was 2.75 with the grandfather coded as affected and the grandmother, as unaffected, and 2.45 with both grandparents coded with unknown affectation status. The maximum LOD score in Family B was 2.21. Although both these maxima are below the traditional LOD score requirement of 3 for declaring strong evidence of autosomal linkage [63], this threshold was designed to be conservative, and is actually overly conservative [64, 65]. In addition, with current easy access to sequence data, the original concern about cost of follow-up no longer carries the same concern as it did when the original threshold was proposed.

The SNP markers were checked for genotyping errors using the PLINK [66] and PEDSTATS [67] packages and SNPs with genotyping errors were removed from the analysis. Files were formatted for MCMC linkage analysis and an ideal set of SNPs was chosen for a marker panel with the Pedigree-Based Analysis Pipeline (PBAP) [68], targeting marker spacing of 0.5 centimorgan (cM), minor allele frequency (MAF) > 0.2, and LD between markers < 0.04. Minor allele frequencies (MAFs) for the SNP arrays were based on the 1000 Genomes Project Europeans (http://www.1000genomes.org). Genetic locations (cM) were obtained from the Rutgers Maps, Build 134 [69] to establish marker order. These positions were then converted to positions based on the Haldane map function to comply with the requirements of the analysis methods. Affectation status for the grandparents in Family A was conservatively set to unknown; two additional models, each with one grandparent coded as affected, were run. MCMC-based linkage analysis was conducted with the gl_auto and gl_lods programs of the MORGAN 3.2 package [70–72]. The gl_lods program calculates LOD scores based on the phenotype information, penetrance model, and the inheritance vectors that are estimated by gl_auto for each marker given the available pedigree constellation, the marker data, and the genetic map. For gl_auto, the run conditions were 100,000 total run iterations, 15% burn-in iterations, and 2,000 saved iterations. Chromosomal regions retained for further analysis were required to have LOD_max_ scores > 1. The approximate 95% confidence interval (CI) about the peak was defined as the region between the boundaries about the peak where LOD = LOD_max_− 1 [73].

For CNV analyses in the two families, two sources of input were used. First, genotypes from the exomes were entered into the Copy Number Inference from Exome Reads (CoNIFER) package [74]. For CoNIFER-based CNV discovery, reads from each exome were split into up to two consecutive 36mers and mapped using the single-end mode of mrsFAST [75], then aligned to the hg19 reference genome. Reads per kilobase per million (RPKM) values were calculated and targets with a median RPKM of 1 were excluded. Standardized RPKM values were calculated and a single value decomposition (SVD) algorithm was applied. The output from this analysis, SVD-ZRPKM, was used as the normalized relative copy number of a given exon in a sample. To exclude naturally occurring regions that are duplicated or repeated in the genome, CNVs were filtered using a 50% reciprocal overlap mask. The second source of input for CNV analysis was the set of 16 Illumina HCE/1-0 (Family A) and 14 Illumina HCE/1-1 (Family B) SNP genotypes. Here, we calculated CNVs with two software packages, PennCNV [76] and cnvHap [77]. PennCNV uses a hidden Markov model (HMM) approach, incorporating several types of information including total signal intensity, allelic intensity ratio at each marker, distance between SNPs, and allele frequencies. To avoid biased results, we did not use pedigree information [78]. Like PennCNV, cnvHap uses an HMM approach but additionally incorporates chromosome-wide haplotypic information and cluster-based models of allele frequencies at each marker position. Specifically regarding the previously reported deletion regions on chrs 2 and 16 [26, 35–37], Illumina HCE/1-0 and HCE/1-1 genotypes from two affected members per family were examined for presence of heterozygous genotypes.

In Family A only, IBD analysis was performed using the HCS genotypes and the BEAGLE software package, Version 3.3.2 [79]. The SNP base calls were normalized to the forward genomic reference strand and converted to PLINK [66] format with the participants coded as unrelated. The unphased genotypes of 165 unrelated HapMap3 Caucasians (CEU) were merged by PLINK with the genotypes of the eight participants. Duplicated SNPs and SNPs with inconsistent locations were deleted. The genotypes of the participants and the HapMap3 Caucasians were phased as unrelated subjects in BEAGLE. The fastIBD routine of BEAGLE was then used to estimate the shared haplotype frequencies among all pairs, inputting default parameters. Ten haplotype pairs were sampled for each participant during each iteration of phasing. Very rare shared haplotypes between pairs (a threshold of a fastIBD score of 1.0e-10) are likely to be identical by descent. The results of ten independent FastIBD analyses were combined. Exclusive regions of haplotype sharing unique to affected participants were compared to the results from linkage analyses. Specifically, a region shared exclusively by the six affected grandchildren selected for SNP typing was required to be shared in all 15 pairwise comparisons. To determine IBD sharing with one of the grandparents, the region in question was required to be shared by the grandparent and all six selected grandchildren.

Selected variants in Family A were tested for segregation using single-marker parametric linkage analysis based on the same parameters as the genome-wide multipoint linkage analysis, here using MERLIN [80] with customized bit size to accommodate the pedigree size. This step was repeated for two additional models, one with the grandfather but not the grandmother coded as affected, and one with the reverse affectation assignment.

#### Exome Variant Annotation, Filtering, and Single-Variant Genotyping

Exome variants were annotated using ClinVar (http://www.ncbi.nlm.nih.gov/clinvar) and Seattle Seq 137 (http://snp.gs.washington.edu/SeattleSeqAnnotation137/HelpHowToUse.jsp), Variant Effect Predictor, Release 76 [81, 82], and searched with GEMINI [83]. All DNA physical map locations reported in this study refer to the hg19 reference genome. In the exome sequences, an important filtering criterion was position within regions implicated in linkage analysis. Because of the assumption of autosomal dominant inheritance, heterozygous variant genotypes were prioritized. In Family A, the possibility that the children inherited causal variants from either of the two grandparents was considered. Based on the assumption that the causative change is relatively rare in the population, allele frequencies in control exomes obtained to date by the National Heart, Lung and Blood Institute’s (NHLBI) Exome Sequencing Project (ESP) (http://evs.gs.washington.edu/EVS/) and the 1000 Genomes project for European as well as all populations were consulted to prioritize MAFs of 15% or lower. To maximize reliability, variants with read depths < 10 and variants that failed quality control by GATK [84] were excluded. The average read depth of the retained variants was 73.5. Variants were further evaluated with respect to their functions (e.g., missense, coding-synonymous), using the in-house Genome Variation Server, and predicted functional effects (e.g., benign, possibly damaging), using PolyPhen [85] and the Combined Annotation Dependent Depletion (CADD) scores [86].

Genotyping of selected candidate variants was done using polymerase chain reactions in a thermal cycler (DNA Engine Tetrad 2; MJ Research) followed by Sanger sequencing using an ABI 3130xl DNA Analyzer for capillary electrophoresis and ABI BigDye fluorescent dye terminator cycle sequencing kits (Applied Biosystems, Grand Island, NY). In one case, (*NIPBL* variant), ExoSAP-IT purified PCR products were submitted to Genewiz (Seattle, WA) for Sanger sequencing on ABI 3730xl DNA Analyzers. To obtain genotypes of two variants from *C4orf21*, ExoSAP-IT purified PCR products from all available Family B members were submitted to GenScript (Piscataway, NJ) for sequencing on 3730xl DNA Analyzers.

For exome variant filtering and single-marker linkage analysis, we considered not only variants in the regions with positive evidence for linkage but also variants in previously reported candidate regions for CAS [13–15, 18–24, 26, 35–38] including regions implied in reading/spelling disorders due to reported comorbidities with CAS [9–11]. For exome variant filtering, variants shared by all affected individuals in one or both families were considered most plausible.

### Web Resources

BEAGLE

https://faculty.washington.edu/browning/beagle/b3.html#beaglev4

cnvHap

http://www.imperial.ac.uk/people/l.coin

CoNIFER

http://conifer.sourceforge.net

GEMINI

http://faculty.washington.edu/wijsman/software.shtml

MORGAN

https://www.stat.washington.edu/thompson/Genepi/MORGAN/Morgan.shtml

PBAP (Pedigree Based Analysis Pipeline)

http://faculty.washington.edu/wijsman/software.shtml

PEDSTATS

http://csg.sph.umich.edu/abecasis/Pedstats/download/

PennCNV

http://penncnv.openbioinformatics.org/en/latest/

PLINK

http://pngu.mgh.harvard.edu/~purcell/plink/download.shtml

## Results

### Linkage Analysis

**Fig 3:**
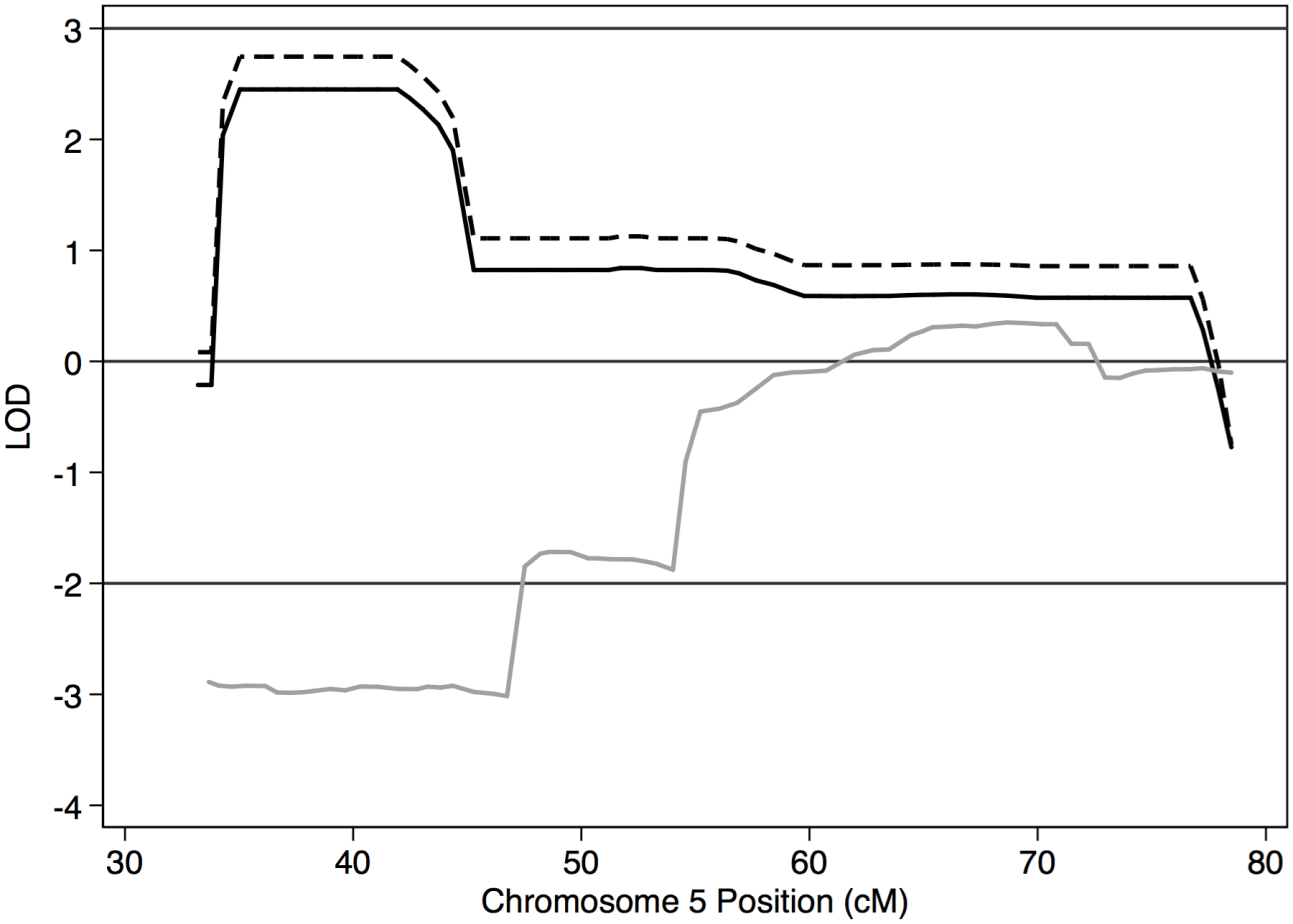
Linkage results for chr 5. Solid black line = Family A with both grandparents coded as affectation unknown; dashed black line = Family A with grandfather coded as affected; solid gray line = Family B.

**Fig 4:**
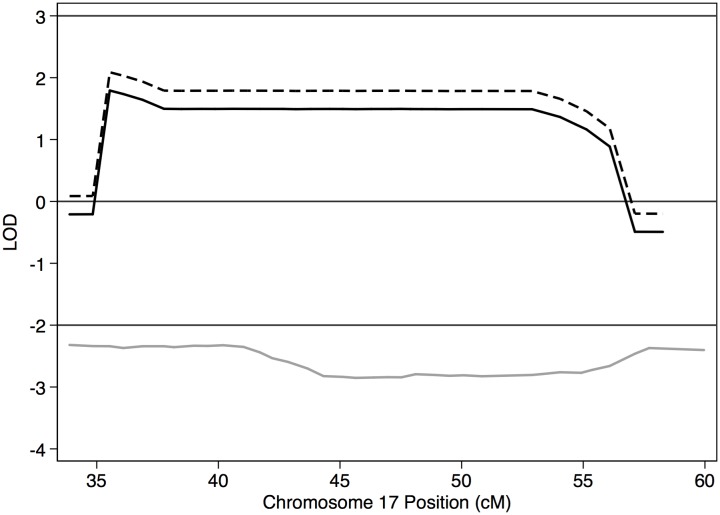
Linkage results for chr 17. Solid black line = Family A with both grandparents coded as affectation unknown; dashed black line = Family A with grandmother coded as affected; solid gray line = Family B.

In Family A, MCMC linkage analysis using PBAP and MORGAN resulted in two regions of interest with peak LOD scores > 1 (Table 1 and Figs 3 and 4). With both grandparents coded as having unknown affectation status, a region of interest with LOD_max_ = 2.45, the maximum score possible under this model, was seen at the 95% CI region of 5p15.1-p14.1 (bp 15,117,438–24,806,593), containing 60 genes. When the linkage analysis for this chromosome was run under the assumption that the grandfather but not the grandmother was affected, consistent with the IBD results described below, LOD_max_ increased to 2.75, again the estimated maximum score given this model. When both grandparents were coded with unknown affectation status, evidence for linkage with LOD_max_ = 1.79 was found at the 95% CI region of 17p13.1-q11.1 (bp 12,582,787–28,550,814), where 341 genes are located. When the linkage analysis was run under the assumption that the grandmother was affected but not the grandfather, as supported by the IBD results for this region, LOD_max_ increased to 2.09. S1 Fig shows linkage results for all autosomes in both families.

**Table 1:** Regions of interest with LOD > 1 in both families.

Fam.	Chr.	Phys. Pos.	Band	cM	LOD_max_	# Genes in Region	95% CI (bp)	95% CI (Band)	cM	# Genes in CI Region
A	5	15,117,438–63,394,447	p15.1-q12.3	34.3–77.2	2.45 (2.75*)	340	15,117,438–24,806,593	p15.1-p14.1	34.3–44.4	60
A	17	12,582,787–28,550,814	p13.1-q11.1	35.5–56.1	1.79 (2.09**)	341	12,582,787–28,550,814	p13.1-q11.1	35.5–56.1	341
B	1	95,781,217–154,626,705	p21.3-q21.3	118.8–149.9	2.02	779	99,733,964–109,352,915	p21.1-p13.2	121.7–129.3	79
B	4	109,931,951–131,629,960	q25-q28.3	112.4–128.1	1.97	186	111,267,671–131,013,140	q25-q28.2	113.8–128.1	163
B	4	155,182,784–175,836,048	q31.3-q34.1	149.7–169.9	1.46	156	162,595,918–175,443,156	q32.1-q34.1	155.6–169.1	100
B	6	138,299,093–147,799,122	q23.3-q24.3	140.8–148.3	1.15	76	138,599,285–147,799,122	q23.3-q24.3	142.5–148.3	73
B	10	32,584,506–56,662,603	p11.22-q21.1	60.2–74.3	1.42	276	32,584,506–54,037,188	p11.23-q11.22	60.2–71.8	266
B	12	101,877,346–115,526,747	q23.2-q24.21	115.1–135.5	1.98	193	104,311,731–105,281,654	q23.3-q24.11	117.7–120.3	15
B	13	19,263,735–23,953,924	q11-q12.12	0–11.91	1.87	101	19,263,735–23,953,924	q11-q12.12	0–10.5	101
B	17	75,839,026–80,986,540	q25.3	130.7–143.8	1.44	143	75,839,026–80,986,540	q25.3	130.7–143.8	143
B	21	14,827,698–16,611,077	q11.2	5.4–10.2	2.03	40	14,827,698–16,611,077	q11.2	5.4–10.2	40

* Family A grandfather coded as affected

** Family A grandmother coded as affected

In Family B, linkage analysis resulted in 9 linkage regions with LOD > 1 (Table 1, S1 Fig). The regions on chrs 1, 4 (q25-q28.3), 12, 13, and 21 are more plausible than the other regions based on the size of the region and LOD scores approaching the estimated maximum scores in this family.

Regions with LOD scores ≤ -2 are considered exclusionary, under the assumption of the correct penetrance mode. Several regions and genes previously identified as loci of interest for CAS overlapped with such regions, as follows: *DCDC2* (Family B), *KIAA0319* (Family B), part of the previously reported 6p21.2-p12.3 region of interest [40] (Family B), *FOXP2* (Family B), *CNTNAP2* (both families), part of the previously reported 7q36.1-q316.3 region of interest [40] (both families), *GALT* [20] (both families), part of the 16p11.2 deletion region [35–37] (Family A), and *CNTNAP1* [38] (both families). Several regions of interest in one family coincided with LOD scores ≤ -2 in the other family (S1 Fig).

### Copy-Number Variation (CNV)

In Family A, CNV analysis based on the four exomes using CoNIFER [74] and the HCE/1-0 SNP array genotypes obtained from the same four samples and 12 additional ones, using PennCNV [76] and cnvHap [77] did not result in any deletions or duplications that segregated with the speech disorder. In the HCE/1-0 SNP genotypes from two affected family members, A-304 and A-311, the regions previously reported deleted in individuals with CAS on 2p16.1 [26] and 16p11.2 [35–37] contained 33.3% and 46.7% heterozygous genotypes, respectively, in the 2p16.1 region and 5.7% and 10.9%, respectively, in the 16p11.2 region.

In Family B, the 15q26.3 duplication encompassing the *FAM169B* gene that had been previously identified in the proband and his mother was confirmed with our probes as well as with the PennCNV, cnvHap, and CoNIFER procedures. The probes and results from PennCNV and cnvHap also identified this duplication in B-202 (affected), B-206 (affectation unknown), and B-301 (unaffected) but not in B-204 (affectation unknown) and B-311 (affected). The second microdeletion involving part of the *MEF2A* gene was not confirmed with any of our CNV methods.

No CNVs shared by the affected family members were identified with PennCNV, cnvHap, or CoNIFER. HCE/1-0 SNP genotypes from two affected participants, B-403 and B-311, contained 41.2% heterozygous genotypes in the *BCL11A* region. In the 16p11.2 region, they contained 4.5% and 11.9% heterozygous genotypes, respectively.

### Identity-by-Descent (IBD) Analysis

In Family A, regions shared IBD as estimated with BEAGLE were observed only in two regions, 5p15.2-p14.1 and 17p12-q11.2. In these regions, there was IBD-sharing by all 15 pairwise comparisons of the six grandchildren selected for HCS SNP genotyping and simultaneously one of the grandparents. The region on chr 5 mutually shared IBD by all 6 grandchildren ranged from bp 14,776,897 to 25,340,617, a region nearly identical to the 95% CI region of interest obtained with linkage analysis. Pairwise comparisons between each of the grandparents and the six grandchildren resulted in matches between the grandfather and each of the six grandchildren between bp 3,265,689 and 25,390,490, and no matches between the grandmother and any of the six grandchildren. On chr 17, pairwise IBD sharing among the six selected grandchildren showed a region common to all 15 pairs, ranging from bp 12,348,755 to 29,783,244, again nearly identical to the results from linkage analysis. The grandfather shared this region with none of the grandchildren but the grandmother shared a region ranging from bp 9,614,556 to 29,943,842 with all six grandchildren.

### Exome Variants in Regions of Interest Based on Linkage Analysis

In Family A, the 95% CI region of interest on chr 5 contained 15 variants, of which the grandfather carried three in the heterozygous state (S1 Table). Of these, the only variant shared exclusively by the grandfather and both grandchildren was rs17285716 in *CDH18* (Cadherin 18 Type 2, OMIM #603019) (Table 2). This variant has a MAF of 0.18 in 1000 Genomes Europeans (0.10 in all populations combined) and a CADD score of 9.71. Two common variants carried by the grandfather, rs11750538 in *MYO10* (Myosin X, OMIM #601481) and rs162848 in *FAM134B* (Family with Sequence Similarity 134, Member B, OMIM #613114), were shared not only by both grandchildren but also the grandmother; the *MYO10* variant was additionally shared by B-311. The extended region with non-negative LOD scores (Table 1) contained nine additional variants with MAF ≤ 0.15 that were exclusively shared by the grandfather and the two grandchildren. In addition, one rare variant located in the *IL7R* gene (Interleukin 7 receptor, OMIM *146661) was shared by the grandfather, the two grandchildren, and all three exomes in Family B. Three variants shared by the grandfather and the two grandchildren were also seen in one or two of the Family B exomes. In the entire chr 5 region of interest, no variants were seen exclusively in the grandmother and the two grandchildren.

**Table 2:** Exome variants in the regions of interest based on linkage analysis results in 95%CI regions and regions with strongest evidence.

Fam.	Chr.	Gene	rs ID	hg19 Position	MAF (1KG EUR)	MAF (1KG All)	CADD	Carrier in Other Family
A*	5	*CDH18*	rs17285716	19,591,174	0.18	0.1	9.71	
A**	17	*NCOR1*	rs61754982	16,004,888	<0.01	<0.01	4.79	
A**	17	*FLCN*	rs3744124	17,124,815	0.03	0.1	0.08	
A**	17	*TOM1L2*	rs143069395	17,786,070	<0.01	<0.01	8.65	
A**	17	*EPN2*	rs55883526	19,216,576	0.01	0.01	1.11	
A**	17	*KSR1*	rs2293180	25,909,816	0.12	0.17	9.37	
A**	17	*TNFAIP1*	rs145418568	26,671,614	0.01	<0.01	0.15	
A**	17	*RPL23A*	rs2288595	27,052,358	0.07	0.11	1.59	
A**	17	*NEK8*	rs200972000	27064924	<0.01	<0.01	15.47	
B	1	*VCAM1*	rs3176878	101,203,698	0.15	0.13	0.01	102, 312
B	1	*COL11A1*	rs17127270	103,405,892	0.18	0.12	0.38	101. 304
B	4	*C4orf21*	rs76187047	113,506,711	0.01	0.02	25.30	
B	4	*C4orf21*	rs61745597	113,544,993	0.01	0.02	7.57	
B	4	*SYNPO2*	rs61732241	119,952,955	0.11	0.04	8.81	101, 304
B	4	*PRDM5*	rs12499000	121,706,201	0.27	0.14	10.32	
B	4	*KIAA1109*	rs6848868	123,150,286	0.08	0.03	10.64	312

* Consistent with inheritance from the grandfather

** Consistent with inheritance from the grandmother

Within the 95% CI region of interest on chr 17, the grandmother carried 111 variants in the heterozygous state. Of these, 7 variants had MAF ≤ 0.15 and were shared with both grandchildren but not the grandfather. None of these 7 variants were found in Family B. In this region of interest, no variants were shared by the grandfather and the grandchildren but not the grandmother.

In Family B, within the 95% CI regions of interest, 40 variants had MAF ≤ 0.15. Of these, 16 were shared only by the three Family B exomes; none segregated in the exomes of both families. Also of these 40 rare variants, seven were located in one of the more compelling regions of interest, which was 4q25-q28.2 for five of them and 1p21.1-p13.2 for the other two (Table 2). Two of the chr 4 variants were rs76187047 and rs61745597, both located in the *C4orf21* gene (Chromosome 4 Open Reading Frame 21, no OMIM number) and not found in Family A. An alias for *C4orf21* is *ZGRF1* (Zinc finger, GRF-type containing 1). The rs76187047 variant is a missense variant with a high CADD score of 25.3. It occurs in <1% of Europeans and all populations. The other *C4orf21* variant is equally rare, also has a missense function, and has a CADD score of 7.6. The rs61732241 variant in the *SYNPO2* gene (Synaptopodin-2, no OMIM number) on chr 4 is not extremely rare and was also seen in two Family A exomes. The rs12499000 variant in the *PRDM5* gene (PR Domain-containing protein 5, OMIM #614161) was only seen in the Family B exomes; it is not rare in Europeans. The rs6848868 variant in the *KIAA1109* gene (OMIM *611565) was also seen in one of the Family A grandchildren. The two chr 1 variants were only rare in all populations combined and also shared by a subset of the Family A exomes. Table 2 lists segregating variants in the 95% CI in the most compelling regions based on linkage analysis. S1 Table lists all segregating variants in the regions of interest.

### Exome Variants in Other Regions of Interest

In 17 previously reported regions or genes of interest for CAS [13–15, 18–24, 26, 35–38], variants with MAF ≤ 0.15 shared by one grandparent and both grandchildren in Family A or all three exomes in Family B were only seen in the 6p21.2-p12.3 region of interest, the 7q11.23 duplication region, the 7q36.1-q38.3 region of interest, and the Senotaxin (*SETX*; OMIM # 608465) gene on chr 9. Most of the variants in 6p21.2-p12.3 were seen in the Family B exomes, whereas the variants in the 7q11.23 duplication region were seen in Family A. One variant, rs386701097 in the Polycystic and Hepatic Disease 1 (*PKHD1*; OMIM # 606702) gene, was seen in all exomes except the grandmother in Family A. S2 Table lists variants segregating in the exomes of either of the two families and carriers in the other family.

### Selected Candidate Variants

In the single-marker linkage analysis in Family A, the highest LOD score, 2.45, was obtained for rs17285716 in the *CDH18* gene in the 95% CI region of interest on chr5p15.1-p14.1 when both grandparents were coded as unknown affectation status and 2.75 when the grandfather was coded as affected (Table 3). A *MYO10* variant in the 5p region of interest had a LOD score of 1.24 regardless of the affectation coding of the grandparents. A variant in the *NIPBL* gene (Nipped-B-Like; OMIM *608667) in the extended 5p region of interest had a LOD score of 1.14 when the grandfather was coded as affected. The second highest LOD score, 1.49, was obtained for two variants on chr 17, rs61754982 (Nuclear Co-receptor Repressor 1, *NCOR1*; OMIM #600849) and rs3744124 (Folliculin, *FLCN*; OMIM #607273), with an increase to LOD = 1.79 when the grandmother, who carries the variants, was coded as affected. Also in the linkage region on chr 17 and inherited from the grandmother, an unannotated variant at position 27,064,924 in the *NEK8* gene (Never in Mitosis A-Related Kines 8, OMIM #609799) had a LOD score of 1.20, increasing to 1.50 when the grandmother was coded as affected. Variants in the *SMCR8* gene (Smith-Magenis Syndrome Chromosome Region, candidate 8; no OMIM #) and the *GLP2R* (Glucagon-Like Peptide 2 Receptor, OMIM #603659)) gene also showed increased LOD scores when the grandmother was coded as affected. Two variants from genes outside the regions of interest, Ankyrin Repeat Domain 12 (*ANKRD12*, OMIM # 610616) and Calcium Channel, Voltage-Dependent, L Type, Alpha-1C subunit (*CACNA1C*, OMIM #114205), also showed increased LOD scores > 1 when the grandmother or the grandfather were coded as affected, respectively. The *ANKRD12* variant has predicted downstream effects and the *CACNA1C* variant is synonymous. The average number of risk alleles across these 10 variants in the affected and unaffected members, respectively, was 11.25 (range: 9,12) and 4.8 (range: 3, 7), not counting the grandparents and the obligate carrier.

**Table 3:** Family A LOD scores from single-marker analysis for variants with LOD > 1. See S3 Table for all tested markers.

Chr. Band	Gene	rs ID	CADD	hg19 Position	Rationale	LOD (Grandp. Aff. Unknown)	LOD (Grandf. Aff.)	LOD (Grandm. Aff.)
5p15.1	*MYO10*	rs396514	8.27	16,794,916	Linkage ROI	1.24	1.24	1.24
5p14.3	*CDH18*	rs17285716	9.71	19,591,174	Linkage ROI; exomes	2.45	2.75	0.88
5p13.2	*NIPBL*	NA	10.98	37,064,663	Linkage ROI	0.87	1.14	-0.09
12p13.33	*CACNA1C*	rs216008	10.35	2,721,137	Cand. gene for dev. dis.	0.90	-0.23	1.19
17p13.1	*GLP2R*	rs1113915	(intron)	9,770,685	Linkage ROI	1.06	-0.42	1.35
17p11.2	*NCOR1*	rs61754982	4.79	16,004,888	Linkage ROI; exomes	1.49	-0.03	1.79
17p11.2	*FLCN*	rs3744124	0.08	17,124,815	Linkage ROI; exomes	1.49	-0.03	1.79
17p11.2	*SMCR8*	rs8080966	9.13	18,220,674	Linkage ROI	0.96	-0.31	1.25
17q11.2	*NEK8*	NA	15.47	27,064,924	Linkage ROI	1.20	-0.28	1.50
18p11.22	*ANKRD12*	rs116726679	0.60	9,255,539	Dyslexia cand. gene	1.09	1.38	-0.34

In Family B, the *C4ord21* variants, both of which are rare and deleterious, were linked and found in all affected family members, one unaffected member, and three members of unknown affectation, one of whom, B-405, had a history of difficulties with written language. The average number of risk alleles among the affected and unaffected members, respectively, was 2 (no variation) and 0.4 (range: 0, 2).

Table 3 summarizes the results from Sanger genotyping and single-marker linkage analysis for all markers with single-marker LOD scores > 1 in Family A. S3 Table shows results for all tested markers. Tables 4 and 5 summarize the number of risk alleles in the Family A and B members, respectively, by variant and affectation status.

**Table 4:** Number of risk alleles in Family A members.

ID	Aff.	*MYO10*	*CDH18*	*NIPBL*	*CACNA1C*	*GLP2R*	*NCOR1*	*FLCN*	*SMCR8*	*NEK8*	*ANKRD12*
		rs396514	rs17285716	NA	rs216008	rs1113915	rs61754982	rs3744124	rs8080966	NA	rs116726679
101	(GF)	1	1	1	0	0	0	0	1	0	1
102	(GM)	1	0	0	1	2	1	1	2	1	0
206	Aff.	1	1	1	1	1	1	1	1	1	1
207	Aff.	1	1	1	1	1	1	N/A	1	1	1
304	Aff.	1	1	1	1	1	1	1	1	1	1
305	Aff.	1	1	1	1	1	1	1	1	1	1
310	Aff.	1	1	1	1	2	1	1	1	N/A	1
311	Aff.	1	1	0	1	1	1	1	1	1	1
312	Aff.	1	1	1	1	2	1	1	1	1	1
314	Aff.	1	1	1	2	1	1	1	2	1	1
202	Unaff.; OC	0	1	1	1	1	1	N/A	2	1	1
201	Unaff.	1	0	0	1	0	0	0	0	0	1
204	Unaff.	1	0	0	0	1	1	1	1	1	1
205	Unaff.	2	0	0	1	1	0	0	0	0	0
208	Unaff.	2	0	0	1	0	0	0	1	0	0
303	Unaff.	1	0	0	0	0	1	1	1	1	1
309	Unaff.	2	0	0	1	2	0	0	0	0	0
302	Unk.	0	0	0	1	0	0	N/A	1	0	2
313	Unk.	2	0	0	2	2	1	1	1	1	1

**Table 5:** Number of risk alleles in Family B members.

ID	Affectation	*C4orf21*	*C4orf21*
		rs61745597	rs76187047
404	Aff.	1	1
302	Aff.	1	1
409	Aff.	1	1
311	Aff.	1	1
303	Unaff.	0	0
205	Unaff.	0	0
505	Unaff.	1	1
410	Unaff.	0	0
301	Unaff.	0	0
308	Unaff.; OC	1	1
204	Unkn.	1	1
206	Unkn.	0	0
405	Unk. OC	1	1
202	Unk. OC	1	1

## Discussion

The purpose of this study was to investigate the genetic etiology of a severe form of speech sound disorder, childhood apraxia of speech (CAS), in two multigenerational families. We used complementary approaches and compared the results from the two families to each other and to previously described findings in individuals with CAS. Results are consistent with different genetic etiologies in the two families as well as a heterogeneous etiology more broadly, because previously reported candidate genes were not confirmed in either of the two families.

In Family A, linkage analysis resulted in two regions of interest located at 5p15.1-p14.1 and 17p13.1-q11.1, both of which overlapped partially with regions that provide exclusionary evidence in Family B. The results from linkage models with one or the other of the grandparents coded as affected, IBD testing, exome variant analysis, and genotyping the candidate variants in the whole family suggest that affected individuals in the family inherited the 5p15.1-p14.1 region from the grandfather and the 17p13.1-q11.1 region, from the grandmother. According to the family interviews, the grandfather but not the grandmother had undergone speech therapy as a child, although both had biological relatives with difficulty in the area of speech and language. It is therefore plausible to suspect that the region of interest on chr 5 harbors one or more variants influencing speech development, whereas the region of interest on chr 17 may harbor variants influencing other inherited traits in the family or may represent a false positive result. Alternatively, it is possible that variants in the 17p13-q11.1 region or both of these regions influenced speech development in the affected family members. The comparison of risk alleles in both regions and additional candidate loci in the affected and unaffected members of the family shows that the affected group had more than twice as many risk alleles as the unaffected group. This finding is consistent with multiple factors influencing the phenotype, possibly with additive effects, similar to findings in other neurological disorders. The unaffected obligate carrier had the risk allele of most of these loci; the reasons for her lack of speech difficulties are not clear.

In the single-marker linkage analysis, the highest obtainable LOD score of 2.75, as estimated by power analysis with one grandparent coded as affected, was seen for only one tested variant, rs17285716 in the *CDH18* gene located in the 5p15.1-p14.1 region of interest. This variant is synonymous but has a high conservation score based on 100 vertebrates basewise conservation score by Phylop and a high GERP score based on 35 mammalian alignments, and its scaled CADD score of 9.71 places it close to the top ten percent of variants in pathogenicity. Synonymous variants have been implicated in other disorders, for instance in a parkinsonian disorder where a synonymous variant was associated with exon skipping [87]. *CDH18* is specifically expressed in the central nervous system and is thought to influence synaptic adhesion, axon outgrowth, and axon guidance, thus regulating the development of the central nervous system [88–90]. The Allen Human Brain Atlas (AHBA; http://human.brain-map.org/) [91] shows maximum *CDH18* expression levels throughout the cerebellar cortex. A DECIPHER (https://decipher.sanger.ac.uk) [92] search yielded 44 syndromic cases with CNVs involving *CDH18* including a case with apraxia, speech and language development, hyperactivity, intellectual disability and a 2.95 Mb duplication partially involving *MY010* and *CDH18*, and a case with attention deficit hyperactivity disorder, speech apraxia, and a 6.96 Mb deletion ranging from *DNAH5* to part of *CDH18*. Speech and language phenotypes were noted in two additional cases with *CDH18* CNVs.

It is possible that *CHD18* influences speech development by acting in concert with other, functionally related genes via regulatory mechanisms. The functional network of *CDH18* includes 15 cadherin genes (*CDH1* through *CDH13*, *CDH17*, and *CDH24*), all influencing cell adhesions, as well as three cadherin-associated protein genes, *CTNNA1*, *CTNNB1*, and *CTNND1*); four of these genes are expressed in the cerebellum. The 5p region of interest harbors two of the cadherin genes, *CDH12* (OMIM #600562) and *CDH10* (OMIM #604555), as well as the *MYO10* gene, where one variant had a single-marker LOD score of 1.24 regardless of affectation status of the grandparents, both of whom are carriers. *MYO10* encodes a protein that belongs to the myosin superfamily and is expressed in epithelia-rich tissues [93]. Its protein product is expressed in many tissue types; in the central nervous system, the AHBA shows high gene expression levels in the basal ganglia and the thalamus. *MYO10* plays a role in axon development, neurite outgrowth, and radial neuron migration in the developing cortex and cell-matrix adhesion [94]. One variant in the *NIPBL* gene on 5p13.2 has no dbSNP rs number and is extremely rare in the population, found neither in the deep population sequencing ESP (n = 6,503) nor in 1000 Genomes. *NIPBL* codes for a protein necessary for the cohesion of sister chromatids during mitosis [95] and is disrupted by some translocations in Cornelia-de-Lange syndrome [96]. This gene is expressed in many tissue types including brain. The AHBA shows maximum expression levels in the basal ganglia, cerebellum, and corpus callosum.

Additional support for the 5p15.1-p14.1 region of interest is found in the autism literature, where common variants in this region have been implicated [97, 98]. Autism spectrum disorder and CAS co-occur in proportions of cases greater than expected by chance [99].

The second highest single-marker LOD score, 1.79, was seen for two variants in the 17p13.1-q11.1 region of interest with the grandmother coded as affected, rs61754982 in *NCOR1* and rs3744124 in *FLCN*. *NCOR1* is involved in thyroid hormone and retinoid acid repression [100]. The AHBA shows maximum expression levels in the basal ganglia and the cerebellar cortex. DECIPHER lists 20 syndromic cases with CNVs involving *NCOR1* including one with truncal ataxia, one with apraxia, and one with delayed speech and language development. *FLCN* mutations are implicated in Birt-Hogg-Dube syndrome [101], a disease involving fibrofolliculomas, renal tumors, lung cysts, and pneumothorax. The AHBA shows maximum expression levels in the dentate gyrus and the cerebellar cortex. DECIPHER lists 92 syndromic cases with CNVs involving *FLCN* including 10 cases with delayed speech and language development. Also in the linkage region on chr 17 and inherited from the grandmother, an unannotated variant in the *NEK8* gene had a LOD score of 1.50 when the grandmother was coded as affected. *NEK8* is expressed in various brain regions including cerebellar nuclei and is thought to play a role in fetal organ development and polycystic kidney disease [102]. A variant within the *SMCR8* gene in the Smith-Magenis Syndrome region was also inherited from the grandmother. This syndrome is characterized by a diverse set of traits including mild intellectual disability, delayed speech and language abilities, distinctive facial features, abnormal sleep patterns, and challenging behaviors. Only the speech trait fits the phenotypic profile observed in Family A. Also inherited from the grandmother was a variant in the *GLP2R* (glucagon-like peptide 2 receptor) gene, which plays a role in intestinal growth and nutrient absorption. According to the AHBA, *SMCR8* expression is widely dispersed throughout regions of the brain whereas *GLP2R* is mainly expressed in cortical regions. Of five cases with CNVs involving *GLP2R* listed in DECIPHER, a 2.45 Mb deletion was associated with autism and delayed speech and language development.

Single-marker linkage analysis resulted in LOD scores > 1 for variants in two genes outside the regions of interest from linkage analysis. *ANKRD12* is located within a dyslexia candidate region, DYX6, on 18p11.22. It is expressed in many tissues including brain [103], most strongly in the cerebellar cortex according the AHBA. A single-point LOD score of 1.38 when the grandmother was coded as affected was obtained for rs116726679 within this gene. This variant is synonymous with predicted downstream effects. *CACNA1C* is involved in cellular processes including contraction and electric signaling [104]. Channelopathies associated with *CACNA1C* have been observed in psychiatric disorders [105], although these were not reported for Family A. According to the AHBA, strongest expression levels are in the thalamus. This variant is synonymous. A LOD score >1 was only seen when the grandmother was coded as affected.

In Family B, the two most plausible exome variants, rs61745597 and rs76187047, are located in the *C4orf21* (*ZGRF1*) gene. These highly deleterious variants are linked and carried by all affected members, the three obligate carriers (two with unknown affectation and one unaffected), and only one other unaffected member; they were not found in four unaffected members and one member with unknown affectation. One carrier with unknown speech affectation status had a history of written language difficulties, raising the possibility of variable expressivity. *C4orf21* is not yet well characterized in terms of function and functional networks. One paralogous protein is encoded by *SETX*, one of the genes of interest for CAS [38]. Although *SETX* is not located within a region with positive evidence for linkage, the grandfather and the two grandchildren in Family A share a rare variant in this gene. *SETX* is associated with autosomal recessive spinocerebellar ataxia-1 and ataxia-oculomotor apraxia-2 [106]. *C4orf21* may encode similar functions related to motor praxis. Similar to the most plausible genes in Family A, *C4orf21* is highly expressed in the cerebellum. Of 10 cases with CNVs involving this gene listed in the DECIPHER database, one, a 23 Mb deletion, is characterized by craniosynostosis and delayed speech and language.

Because *CDH18*, several functionally related genes, *C4orf21*, and other genes of interest in both families are strongly expressed in the cerebellum, it is possible that genetic influences converge on the cerebellum, producing downstream effects on speech and other behaviors. The cerebellum plays a crucial role not only in complex motor processes but also in linguistic and cognitive activities, observable in infants during speech perception [107] and in children and adults during tasks requiring sequential processing such as applying syntactic rules [108, 109]. Cerebellar anomalies were implicated in functional and structural imaging studies of the KE family with severe CAS caused by a *FOXP2* mutation [16, 17]. The deficits in motor sequencing as well as those in linguistic and cognitive tasks observed in our CAS studies [39, 42, 52] are consistent with cerebellar involvement, although brain imaging data were not collected.

Only two variants, rs2228141 in the *IL7R* gene, located in the 5p region of interest but outside the 95% CI, and rs386701097 in the *PKHD1* gene in a region previously reported as a region of interest, were shared by all relevant samples in both families (one grandparent and both grandchildren in Family A and all Family B exomes). *IL7R* plays a role in the immune system and is implicated in multiple sclerosis, whereas *PKHD1* influences kidney and liver functions. It is uncertain whether these variants contributed to the phenotype.

We found no evidence of causal CNVs in either of the families. In Family B, one region of a previously identified duplication on 15q was confirmed in the proband and his mother, but was not found in an affected family member, making it unlikely to be causal in this family. CNVs occur frequently without any pathogenic effects [110]. In the two families, we found no evidence for CNVs that had previously been reported to associate with CAS [23, 26, 35, 36], and we also effectively ruled out deletions in the 2p16.1 and 16p11.2 regions by showing heterozygous genotypes in affected family members of both participating families in these regions. Similarly, we found no evidence for most of the previously reported validated or candidate single nucleotide variations [13, 14, 38]. Exceptions are the *SETX* variant in Family A, variants in regions of interest in 6p21.2-p12.3 (Family B), 7q11.23 and 7q36.1-q36.3 (Family A). Whether or not these variants contributed to the phenotype is uncertain.

Taken together, our results are consistent with the hypothesis that the CAS phenotype, like many other neurological phenotypes including Alzheimer’s disease and autism spectrum disorder, is a complex and genetically heterogeneous disorder with several discoverable variants, each of which segregates and confers risks of varying levels of impact. The authors of a recent study of an extended family with Alzheimer’s disease report multiple segregating risk factors of high impact including *ApoE4* and *TREM2*, where the effects of the variants were interpreted as additive [111]. Multiple-hit risks for autism spectrum disorder have been observed in a sporadic case [112]. In simplex cases and multigenerational families with autism spectrum disorder, imbalances in multiple genes were found to contribute to the disease state [113]. We posit that we discovered at least one variant of high impact in each of the two families but that there may be other factors influencing the trait. Whether or not distinctly different genetic etiologies have clinical implications for diagnosis and therapy remains to be investigated.

Ongoing efforts to characterize the genetic etiology of CAS and other forms of speech sound disorders will lead to early identification of infants at genetic risk and motivate the development of effective preventative measures. Detailed knowledge of genotype-phenotype associations will also provide the basis for subtype-specific, customized therapy approaches.

## Limitations and Future Directions

Learning to speak is a complex task thought to be influenced by many variables related to genes and environment. Additional sources of variability in the phenotype of the affected family members are their relative ages and type and duration of intervention. It is possible that individuals who married into the family contributed additional genetic risk factors that modified the speech development in the children, potentially adding genetic heterogeneity.

The assumptions underlying the present study included relatively low allele frequencies, few causal variants, and heterozygous genotypes exclusively shared by the affected family members. If CAS were the result of many variants, each common and of very small effect, then our methodology would be unsuitable to identify them. Similarly, it is possible that one or more causal variants reside outside the exome in regulatory DNA regions, in which case we would have been unable to detect them.

We obtained CNV data from two sources (exomes, SNP array) and three algorithms (CoNIFER, cnvHap, PennCNV). It is possible that some CNVs were missed or reported as false positives.

Future plans include investigating these and other multigenerational families with severe forms of speech sound disorder consistent with CAS using whole genome sequences of all informative family members to look for segregating variants. Such studies have the potential to identify causal variants, even in cases of polygenic etiologies where multiple genes, each of moderate effect, shape the ability to learn to speak. In addition, we will investigate samples from many smaller families for shared variations, as these families would not provide sufficient statistical power for linkage analysis in individual families but pooled together, they would provide useful data toward discovery of contributing variants. Efforts to characterize the genetic of CAS and other forms of speech sound disorders will lead to early identification of infants at genetic risk and motivate the development of effective preventative measures.

## Supporting Information

S1 Fig Linkage analysis results for all autosomes in both families. Previously reported candidate regions are marked along the cM axis.
(TIF)

S1 Table Exome variants in the regions of interest based on linkage analysis results. (DOCX)

S2 Table Segregating exome variants in previously identified CAS genes or regions of interest in Family A. (DOCX)

S3 Table Family A LOD scores from single-marker analysis for all tested variants. (DOCX)
