# Supplementary figures and images for: Genetic Candidate Variants in Two Multigenerational Families with Childhood Apraxia of Speech
Source: PLoS One. 2016 Apr 27;11(4):e0153864. doi: 10.1371/journal.pone.0153864 (PMC4847873; doi:10.1371/journal.pone.0153864)

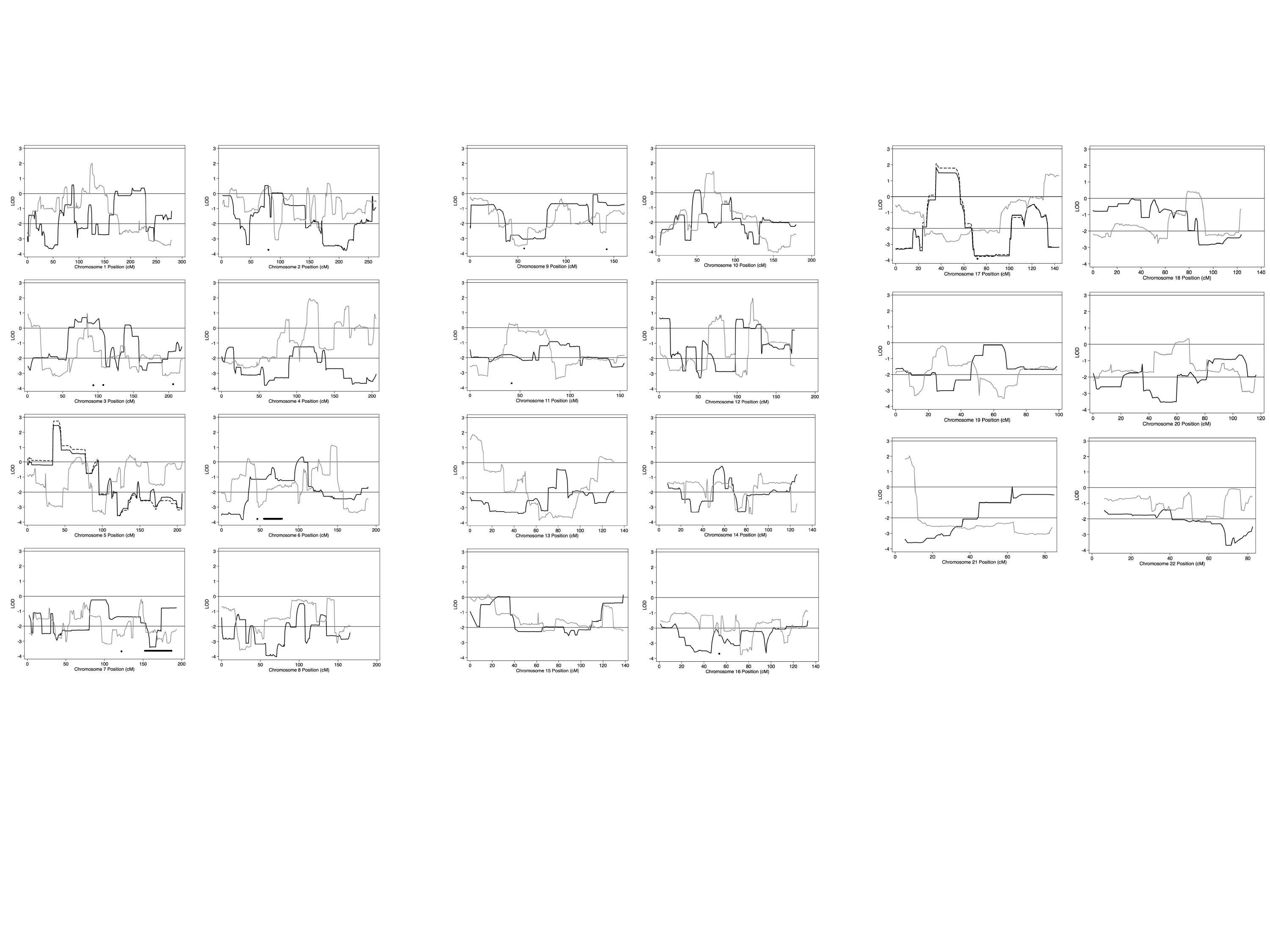

Supplement: S1 Fig — Previously reported candidate regions are marked along the cM axis. (TIF) [file pone.0153864.s001.tif]
