# Supplementary material for: Genetic Candidate Variants in Two Multigenerational Families with Childhood Apraxia of Speech
Source: PLoS One. 2016 Apr 27;11(4):e0153864. doi: 10.1371/journal.pone.0153864 (PMC4847873; doi:10.1371/journal.pone.0153864)
Supplement: S1 Table — (DOCX) [file pone.0153864.s002.docx]

| Fam. | Chr. | Gene | rs ID | hg19 Position | MAF (1KG EUR) | MAF (1KG All) | CADD | Carrier in Opposite Family |
| --- | --- | --- | --- | --- | --- | --- | --- | --- |
| A* | 5 ^CI^ | *CDH18* | rs17285716 | 19,591,174 | 0.18 | 0.1 | 9.71 |  |
| A* | 5 | *MTMR12* | rs61755422 | 32,233,963 | 0.01 | 0.02 | 10.53 |  |
| A* | 5 | *ZFR* | rs4867440 | 32,403,346 | 0.05 | 0.06 | 8.52 |  |
| A* | 5 | *PRLR* | rs2228482 | 35,084,647 | 0.02 | 0.07 | 2.19 |  |
| A* | 5 | *SPEF2* | rs34708521 | 35,670,303 | 0.06 | 0.16 | 21.50 |  |
| A* | 5 | *SPEF2* | rs62351876 | 35,695,915 | 0.06 | 0.21 | 7.09 |  |
| A* | 5 | *IL7R* | rs2228141 | 35,871,273 | 0.14 | 0.13 | 4.15 | 202, 311, 403 |
| A* | 5 | *NIPBL* | None | 37,064,663 | <0.01 | <0.01 | 10.98 |  |
| A* | 5 | *ZNF131* | rs36122541 | 43,162,291 | 0.12 | 0.12 | 0.05 | 403 |
| A* | 5 | *ZNF131* | rs35397154 | 43,175,103 | 0.12 | 0.12 | 2.18 | 403 |
| A* | 5 | *CDC20B* | rs1051846 | 54,404,057 | 0.13 | 0.19 | 4.49 |  |
| A* | 5 | *MIR449B* | rs10061133 | 54,466,544 | 0.1 | 0.12 | 15.91 |  |
| A* | 5 | *ACTBL2* | rs61737336 | 56,778,103 | 0.08 | 0.06 | 2.82 |  |
| A* | 5 | *HTR1A* | rs1799921 | 63,257,465 | 0.01 | <0.01 | 1.40 | 311, 403 |
| A** | 17 ^CI^ | *NCOR1* | rs61754982 | 16,004,888 | <0.01 | <0.01 | 4.79 |  |
| A** | 17 ^CI^ | *FLCN* | rs3744124 | 17,124,815 | 0.03 | 0.1 | 0.08 |  |
| A** | 17 ^CI^ | *TOM1L2* | rs143069395 | 17,786,070 | <0.01 | <0.01 | 8.65 |  |
| A** | 17 ^CI^ | *EPN2* | rs55883526 | 19,216,576 | 0.01 | 0.01 | 1.11 |  |
| A** | 17 ^CI^ | *KSR1* | rs2293180 | 25,909,816 | 0.12 | 0.17 | 9.37 |  |
| A** | 17 ^CI^ | *TNFAIP1* | rs145418568 | 26,671,614 | 0.01 | <0.01 | 0.15 |  |
| A** | 17 ^CI^ | *RPL23A* | rs2288595 | 27,052,358 | 0.07 | 0.11 | 1.59 |  |
| A** | 17 ^CI^ | *NEK8* | rs200972000 | 27064924 | <0.01 | <0.01 | 15.47 |  |
| B | 1+ ^CI^ | *VCAM1* | *rs3176878* | *101,203,698* | 0.15 | 0.13 | 0.01 | 102, 312 |
| B | 1+ ^CI^ | *COL11A1* | *rs17127270* | *103,405,892* | 0.18 | 0.12 | 0.38 | 101. 304 |
| B | 1+ | *CHI3L2* | rs11556868 | 111,778,325 | 0.12 | 0.07 | 19.16 |  |
| B | 1+ | *ADORA3* | rs2275797 | 112,033,285 | 0.05 | 0.15 | 4.12 | 312 |
| B | 1+ | *LCE1C* | rs2006940 | 152,777,908 | 0.11 | 0.22 | 9.62 | 102, 304 |
| B | 4+ ^CI^ | *C4orf21* | rs76187047 | 113,506,711 | 0.01 | 0.02 | 25.30 |  |
| B | 4+ ^CI^ | *C4orf21* | rs61745597 | 113,544,993 | 0.01 | 0.02 | 7.57 |  |
| B | 4+ ^CI^ | *SYNPO2* | rs61732241 | 119,952,955 | 0.11 | 0.04 | 8.81 | 101, 304 |
| B | 4+ ^CI^ | *PRDM5* | rs12499000 | 121,706,201 | 0.27 | 0.14 | 10.32 |  |
| B | 4+ ^CI^ | *KIAA1109* | rs6848868 | 123,150,286 | 0.08 | 0.03 | 10.64 | 312 |
| B | 6 ^CI^ | *TXLNB* | rs72988902 | 139,569,043 | 0.00 | 0.00 | 11.42 |  |
| B | 6 ^CI^ | *FBXO30* | rs386605930 | 146,127,029 | 0.01 | 0.09 | 2.31 |  |
| B | 10 ^CI^ | *ITGB1* | rs2230396 | 33,209,266 | 0.12 | 0.22 | 8.65 |  |
| B | 10 ^CI^ | *ITGB1* | rs2230395 | 33,211,227 | 0.12 | 0.19 | 11.20 | 101 |
| B | 10 ^CI^ | *ITGB1* | rs2230394 | 33,217,110 | 0.12 | 0.20 | 3.27 | 101 |
| B | 10 ^CI^ | *WDFY4* | rs141273838 | 49,951,198 | 0.01 | 0.00 | 1.27 | 102, 304 |
| B | 10 ^CI^ | *WDFY4* | rs41283283 | 50,098,739 | 0.09 | 0.03 | 16.14 | 102, 304 |
| B | 10 ^CI^ | *WDFY4* | rs41302987 | 50,121,454 | 0.07 | 0.03 | 0.94 | 102, 304 |
| B | 12+ | *STAB2* | rs7306642 | 104,139,034 | 0.07 | 0.08 | 0.54 |  |
| B | 12+ | *TMEM119* | rs73191224 | 108,985,785 | 0.06 | 0.05 | 9.87 |  |
| B | 12+ | *SELPLG* | rs2228315 | 109,017,898 | 0.07 | 0.18 | 2.63 |  |
| B | 17 ^CI^ | *CCDC40* | rs60684213 | 78,059,889 | 0.09 | 0.11 | 12.61 | 304 |
| B | 17 ^CI^ | *GAA* | rs1800305 | 78,083,791 | 0.07 | 0.10 | 3.06 |  |
| B | 17 ^CI^ | *ENDOV* | rs41301932 | 78,406,974 | 0.05 | 0.06 | 13.35 | 101, 312 |
| B | 17 ^CI^ | *BAIAP2* | rs138099755 | 79,082,245 | 0.02 | 0.01 | 2.81 |  |
| B | 17 ^CI^ | *BAIAP2* | rs4969391 | 79,089,590 | 0.16 | 0.17 | 13.79 | 101, 312 |
| B | 17 ^CI^ | *NPLOC4* | rs17852306 | 79,580,457 | 0.04 | 0.09 | 2.24 | 312 |
| B | 17 ^CI^ | *HGS* | rs34384005 | 79,662,067 | 0.07 | 0.11 | 7.83 | 312 |
| B | 17 ^CI^ | *HGS* | rs56058441 | 79,668,135 | 0.07 | 0.10 | 2.27 | 312 |
| B | 17 ^CI^ | *SLC25A10* | rs11546280 | 79,671,714 | 0.07 | 0.10 | 0.04 | 312 |
| B | 17 ^CI^ | *GCGR* | rs140065949 | 79,769,834 | 0.03 | 0.02 | 4.42 |  |
| B | 17 ^CI^ | *SIRT7* | rs61752690 | 79,880,565 | 0.00 | 0.00 | 7.93 |  |
| B | 17 ^CI^ | *DUS1L* | rs11542332 | 80,020,812 | 0.05 | 0.13 | 10.65 |  |
| B | 17 ^CI^ | *FOXK2* | rs35832203 | 80,559,272 | 0.03 | 0.01 | 12.61 |  |

* = Variant shared by Fam. A grandfather and both grandchildren

** = Variant shared by Fam. A grandmother and both grandchildren

^CI^ = Located within 95% confidence interval

+ = Located in region of interest with stronger evidence for linkage (Fam. B)
