# Supplementary material for: Genetic Candidate Variants in Two Multigenerational Families with Childhood Apraxia of Speech
Source: PLoS One. 2016 Apr 27;11(4):e0153864. doi: 10.1371/journal.pone.0153864 (PMC4847873; doi:10.1371/journal.pone.0153864)
Supplement: S2 Table — (DOCX) [file pone.0153864.s003.docx]

| Chr | Gene/Region | bp | cM | Gene in Region | rs ID | hg19 Position | MAF (1KG EUR) | MAF (1KG All) | CADD | Fam. A Carrier | Fam. B Carrier |
| --- | --- | --- | --- | --- | --- | --- | --- | --- | --- | --- | --- |
| 2 | *BCL11A* | 60,684,329-60,780,633 | 83 |  |  |  |  |  |  | None | None |
| 3 | *FOXP1* | 71,003,865-71,180,092 | 96 |  |  |  |  |  |  | None | None |
| 3 | *ROBO1* | 78,655,883-78,719,690 | 107 |  |  |  |  |  |  | None | None |
| 3 | *ATP13A4* | 193,119,866-193,272,696 | 208 |  |  |  |  |  |  | None | None |
| 6 | *DCDC2* | 24,171,983-24,383,520 | 48 |  |  |  |  |  |  | None | None |
| 6 | *KIAA0319* | 24,544,332-24,646,383 | 48 |  |  |  |  |  |  | None | None |
| 6 | 6p21.2-p12.3 ROI | 36,632,927-64,590,642 | 55-81 | *DNAH8* | rs45519938 | 38,820,493 | 0.01 | 0.00 | 21.80 | None | 202, 311, 403 |
|  |  |  |  | *DNAH8* | rs112903128 | 38,899,691 | 0.00 | 0.00 | 0.04 | None | 202, 311, 403 |
|  |  |  |  | *TREM2* | rs143332484 | 41,129,207 | 0.01 | 0.00 | 8.31 | None | 202, 311, 403 |
|  |  |  |  | *PGC* | rs4711690 | 41,708,938 | 0.13 | 0.21 | 11.13 | 101, 304, 312* | None |
|  |  |  |  | *TRERF1* | rs2295275 | 42,224,546 | 0.08 | 0.05 | 23.70 | 312 | 202, 311, 403 |
|  |  |  |  | *UBR2* | rs17855250 | 42,573,513 | 0.11 | 0.06 | 9.29 | 304 | 202, 311, 403 |
|  |  |  |  | *UBR2* | rs62414625 | 42,585,022 | 0.11 | 0.06 | 9.03 | 304 | 202, 311, 403 |
|  |  |  |  | *UBR2* | rs5014584 | 42,600,319 | 0.11 | 0.06 | 10.00 | 304 | 202, 311, 403 |
|  |  |  |  | *PRPH2* | rs425876 | 42,666,145 | 0.11 | 0.06 | 8.73 | 304 | 202, 311, 403 |
|  |  |  |  | *CUL7* | rs386700663 | 43,014,298 | 0.03 | 0.01 | 9.24 | 304 | 202, 311, 403 |
|  |  |  |  | *CUL7* | rs386700663 | 43,014,299 | 0.03 | 0.01 | 3.85 | None | 202, 311, 403 |
|  |  |  |  | *CUL9* | rs62417521 | 43,190,135 | 0.08 | 0.04 | 1.44 | 101, 102, 304, 312 | None |
|  |  |  |  | *TJAP1* | rs146174516 | 43,469,358 | 0.01 | 0.00 | 28.80 | None | 202, 311, 403 |
|  |  |  |  | *RSPH9* | rs16896629 | 43,638,636 | 0.06 | 0.13 | 5.96 | None | 202, 311, 403 |
|  |  |  |  | *GPR116* | rs41348953 | 46,824,448 | 0.08 | 0.16 | 8.36 | 101, 312 | 202, 311, 403 |
|  |  |  |  | *GPR116* | rs613870 | 46,826,508 | 0.08 | 0.16 | 7.05 | 101, 312 | 202, 311, 403 |
|  |  |  |  | *GPR116* | rs572248 | 46,826,715 | 0.08 | 0.16 | 0.02 | 101, 312 | 202, 311, 403 |
|  |  |  |  | *GPR116* | rs571247 | 46,826,844 | 0.08 | 0.16 | 0.01 | 101, 312 | 202, 311, 403 |
|  |  |  |  | *GPR116* | rs611779 | 46,826,910 | 0.08 | 0.16 | 0.30 | 101, 312 | 202, 311, 403 |
|  |  |  |  | *GPR116* | rs386601578 | 46,827,126 | 0.08 | 0.16 | 0.01 | 101, 312 | 202, 311, 403 |
|  |  |  |  | *GPR116* | rs386600165 | 46,834,685 | 0.08 | 0.16 | 1.22 | 101, 312 | 202, 311, 403 |
|  |  |  |  | *GPR116* | rs386605056 | 46,856,100 | 0.14 | 0.14 | 0.47 | None | 202, 311, 403 |
|  |  |  |  | *C6orf141* | rs45551532 | 49,518,599 | 0.02 | 0.06 | 12.61 | None | 202, 311, 403 |
|  |  |  |  | *C6orf141* | rs41273692 | 49,519,169 | 0.02 | 0.06 | 9.46 | None | 202, 311, 403 |
|  |  |  |  | *PKHD1* | rs34548196 | 51,484,226 | 0.02 | 0.01 | 11.20 | None | 202, 311, 403 |
|  |  |  |  | *PKHD1* | rs2661488 | 51,491,866 | 0.02 | 0.04 | 0.04 | None | 202, 311, 403 |
|  |  |  |  | *PKHD1* | rs17667728 | 51,512,887 | 0.04 | 0.03 | 9.18 | None | 202, 311, 403 |
|  |  |  |  | *PKHD1* | rs386701097 | 51,586,772 | 0.14 | 0.22 | 5.06 | 101, 304, 312* | 202, 311, 403 |
|  |  |  |  | *PKHD1* | rs148932323 | 51,611,651 | 0.01 | 0.00 | 14.42 | None | 202, 311, 403 |
|  |  |  |  | *GCM1* | rs13200319 | 52,993,002 | 0.13 | 0.07 | 3.31 | None | 202, 311, 403 |
| 7 | 7q11.23 dup. region | 70,597,523-74,789,332 | 83-88 | *NSUN5* | rs35747107 | 72,719,019 | 0.08 | 0.02 | 2.75 | 101, 304, 312* | None |
| 7 | *FOXP2* | 114,055,052-114,333,827 | 121 |  |  |  |  |  |  | None | None |
| 7 | 7q36.1-q36.3 ROI incl. *CNTNAP2* | 143,723,666-159,138,663 | 152-193 | *SSPO* | rs10261977 | 149,528,262 | 0.15 | 0.19 | 4.16 | 101, 304, 312* | None |
|  |  |  |  | *SSPO* | rs1557956 | 149,515,796 | 0.15 | 0.20 | 0.00 | 101, 304, 312* | None |
| 7 | *CNTNPA2* | 145,813,453-148,118,088 |  |  |  |  |  |  |  | None | None |
| 9 | *GALT* | 34,646,586-34,650,595 | 57 |  |  |  |  |  |  | None | None |
| 9 | *SETX* | 135,136,827-135,230,372 | 147 |  | rs34073320 | 135,158,690 | 0.03 | 0.01 | 10.08 | 101, 304, 312 | None |
| 11 | *ELP4* | 31,531,297-31,805,329 | 47 |  |  |  |  |  |  | None | None |
| 11 | *PAX6* | 31,806,340-31,839,509 | 47 |  |  |  |  |  |  | None | None |
| 16 | 16p11.2 del. Region | 29,630,168-30,098,069 | 55-56 |  |  |  |  |  |  | None | None |
| 17 | *CNTNAP1* | 40,834,632-40,852,011 | 73 |  |  |  |  |  |  | None | None |

* Consistent with inheritance from Fam. A grandfather

** Consistent with inheritance from Fam. A grandmother
