# Supplementary material for: Genetic Candidate Variants in Two Multigenerational Families with Childhood Apraxia of Speech
Source: PLoS One. 2016 Apr 27;11(4):e0153864. doi: 10.1371/journal.pone.0153864 (PMC4847873; doi:10.1371/journal.pone.0153864)
Supplement: S3 Table — (DOCX) [file pone.0153864.s004.docx]

| Chr. Band | Gene | rs ID | hg19 Position | Rationale | LOD (Grandp. Aff. Unknown) | LOD (Grandf. Aff.) | LOD (Grandm. Aff.) |
| --- | --- | --- | --- | --- | --- | --- | --- |
| 2p15 | *C2orf74* | rs1729674 | 61,389,737 | Dyslexia cand. region | 0.39 | 0.39 | 0.39 |
| 2p12 | *MRPL19* | NA | 75,882,032 | Dyslexia cand. region | -0.30 | -0.05 | -0.94 |
| 2p12 | *C2orf3* | rs141752962 | 75,899,101 | Dyslexia cand. region | 0.00 | 0.00 | 0.00 |
| 2p11.2 | *TCF7L1* | rs11547160 | 85,536,415 | Dyslexia cand. region | -0.60 | -0.37 | -1.08 |
| 2q11.2 | *NPAS2* | rs2305160 | 101,591,304 | Dyslexia cand. region | 0.00 | 0.00 | 0.00 |
| 3p22.3 | *SUSD5* | rs9637517 | 33,255,592 | Exomes; gene function | -0.38 | -0.24 | -0.58 |
| 3p22.1 | *ULK4* | rs4973986 | 41,841,716 | Exomes; gene function | -1.23 | -1.16 | -1.32 |
| 3p22.1 | *KBTBD5* | rs123509 | 42,733,468 | Exomes; gene function | 0.56 | 0.78 | 0.10 |
| 3p21.31 | *C3orf77* | rs9833423 | 44,283,672 | Exomes; gene function | 0.22 | 0.47 | -0.46 |
| 3p21.31 | *C3orf77* | rs7645375 | 44,283,808 | Exomes; gene function | 0.07 | 0.31 | -0.51 |
| 3p21.31 | *ZNF167* | rs9873604 | 44,612,050 | Exomes; gene function | 0.00 | 0.00 | 0.00 |
| 3p21.31 | *SLC6A20* | rs61731475 | 45,801,393 | Exomes; gene function | 0.58 | 0.85 | -0.27 |
| 3p21.31 | *CELSR3* | rs112843572 | 48,680,470 | Exomes; gene function | 0.58 | 0.85 | -0.27 |
| 3p21.31 | *C3orf71* | rs150858838 | 48,956,257 | Exomes; gene function | -0.38 | -0.14 | -0.95 |
| 3p21.31 | *C3orf18* | rs1034405 | 50,597,092 | Exomes; gene function | 0.58 | 0.85 | -0.27 |
| 3p14.3 | *CACNA2D3* | rs4245920 | 54,961,949 | Exomes; gene function | 0.10 | 0.34 | -0.44 |
| 3p14.3 | *FLNB* | rs116826041 | 58,145,348 | Exomes; gene function | 0.47 | 0.72 | -0.21 |
| 3p14.2 | *C3orf14* | rs114729571 | 62,319,048 | Exomes; gene function | 0.04 | 0.18 | -0.17 |
| 3p14.1 | *ATXN7* | rs1053339 | 63,981,424 | Exomes; gene function | 0.04 | 0.18 | -0.17 |
| 3p12.3 | *ROBO1* | NA | 78,667,090 | Dyslexia cand. gene | -1.02 | -0.89 | -1.21 |
| 5p15.1 | *FAM134B* | rs162848 | 16,478,200 | Linkage ROI | 0.66 | 0.66 | 0.66 |
| 5p15.1 | *MYO10* | rs25901 | 16,673,975 | Linkage ROI | 0.00 | 0.00 | 0.00 |
| 5p15.1 | *MYO10* | rs396514 | 16,794,916 | Linkage ROI | 1.24 | 1.24 | 1.24 |
| 5p14.3 | *CDH18* | rs17285716 | 19,591,174 | Linkage ROI; exomes | 2.45 | 2.75 | 0.88 |
| 5p14.3 | *CDH12* | rs57626343 | 22,178,361 | Linkage ROI | -0.62 | -0.63 | -0.61 |
| 5p13.2 | *NIPBL* | NA | 37,064,663 | Linkage ROI; exomes | 0.87 | 1.14 | -0.09 |
| 5q13.2 | *MAP1B* | rs115657474 | 71,490,953 | Linkage ROI | 0 | 0 | 0 |
| 5q13.2 | *MAP1B* | NA | 71,490,955 | Linkage ROI | 0 | 0 | 0 |
| 6p22.3 | *DCDC2* | rs143313706 | 24,178,840 | Dyslexia cand. gene | 0.00 | 0.00 | 0.00 |
| 6p22.3 | *DCDC2* | rs33943110 | 24,357,796 | Dyslexia cand. gene | -0.60 | -0.37 | -1.08 |
| 6p22.3 | *KIAA0319* | rs4504469 | 24,588,884 | Dyslexia cand. gene | -0.12 | 0.12 | -0.66 |
| 6p22.3 | *KIAA0319* | rs4576240 | 24,596,478 | Dyslexia cand. gene | -0.65 | -0.52 | -0.83 |
| 6p21.31 | *PNPLA1* | rs4713956 | 36,275,458 | Dyslexia cand. region | -0.03 | -1.14 | 0.26 |
| 6p21.31 | *ETV7* | rs2234079 | 36,339,136 | Dyslexia cand. region | -0.75 | -0.75 | -0.75 |
| 6p21.1 | *CUL9* | rs62417521 | 43,190,135 | Exomes; gene function | -1.57 | -1.57 | -1.57 |
| 6p12.1 | *MLIP* | rs4712056 | 53,989,526 | Exomes; gene function | -0.50 | -0.31 | -0.86 |
| 7q11.23 | *STYXL1* | rs8565 | 75,630,274 | Exomes; gene function | 0.00 | 0.00 | 0.00 |
| 7q11.23 | *ZP3* | rs2906999 | 76,069,811 | Exomes; gene function | 0.11 | 0.11 | 0.11 |
| 7q11.23 | *DTX2* | rs12534498 | 76,111,938 | Exomes; gene function | -0.30 | -0.22 | -0.41 |
| 7q36.1 | *CNTNAP2* | NA | 147,926,842 | Language cand. gene | -1.20 | -1.63 | -0.99 |
| 11p15.1 | *SLC6A5* | rs1805091 | 20,648,380 | Exomes; gene function | -0.72 | -0.47 | -1.38 |
| 11p14.3 | *ANO5* | rs7481951 | 22,271,870 | Exomes; gene function | 0.01 | 0.01 | 0.01 |
| 11p14.2 | *ANO3* | rs17243252 | 26,353,811 | Exomes; gene function | -1.25 | -1.25 | -1.25 |
| 11p14.2 | *MUC15* | rs15783 | 26,586,801 | Exomes; gene function | -0.93 | -0.73 | -1.31 |
| 11p14.2 | *SLC5A12* | rs72883299 | 26,718,732 | Exomes; gene function | 0 | 0 | 0 |
| 12p13.33 | *CACNA1C* | rs216008 | 2,721,137 | Cand. gene for dev. dis. | 0.90 | -0.23 | 1.19 |
| 12p13.33 | *CACNA1C* | rs56180838 | 2,760,898 | Cand. gene for dev. dis. | 0.63 | -0.13 | 0.89 |
| 13q31.1 | *SLITRK6* | rs12863734 | 86,370,571 | Exomes; gene function | -0.60 | -1.08 | -0.37 |
| 15q13.1 | *HERC2* | rs75181529 | 28,517,436 | Dyslexia cand. region | 0 | 0 | 0 |
| 16q24.1 | *ATP2C2* | rs118105563 | 84,495,404 | Language cand. gene | 0 | 0 | 0 |
| 17p13.1 | *GLP2R* | rs1113915 | 9,770,685 | Linkage ROI | 1.06 | -0.42 | 1.35 |
| 17p11.2 | *NCOR1* | rs61754982 | 16,004,888 | Linkage ROI; exomes | 1.49 | -0.03 | 1.79 |
| 17p11.2 | *TNFRSF13B* | rs34562254 | 16,842,991 | Linkage ROI; exomes | 0.77 | 0.77 | 0.77 |
| 17p11.2 | *FLCN* | rs3744124 | 17,124,815 | Linkage ROI; exomes | 1.49 | -0.03 | 1.79 |
| 17p11.2 | *SMCR8* | rs8080966 | 18,220,674 | Linkage ROI | 0.96 | -0.31 | 1.25 |
| 17q11.2 | *LGALS9* | rs361497 | 25,970,633 | Linkage ROI | 0.38 | 0.38 | 0.38 |
| 17q11.2 | *NEK8* | NA | 27,064,924 | Linkage ROI | 1.20 | -0.28 | 1.50 |
| 17q11.2 | *SLC6A4* | rs2066713 | 28,551,665 | Linkage ROI | -0.20 | -1.15 | 0.08 |
| 18p11.22 | *ANKRD12* | rs116726679 | 9,255,539 | Dyslexia cand. region | 1.09 | 1.38 | -0.34 |
| 18p11.21 | *POTEC* | rs145408650 | 14,542,979 | Dyslexia cand. region | -0.51 | -0.51 | -0.51 |
| 18q12.1 | *B4GALT6* | rs34683195 | 29,205,591 | Dyslexia cand. region | -0.82 | -0.70 | -0.98 |
| 18q12.1 | *MEP1B* | rs233223 | 29,795,101 | Dyslexia cand. region | 0.08 | 0.35 | -0.81 |
| 18q12.3 | *SETBP1* | rs663651 | 42,456,653 | Dyslexia cand. region | -0.86 | -0.86 | -0.86 |
